# ﻿Phylogenetic classification of arbuscular mycorrhizal fungi: new species and higher-ranking taxa in Glomeromycota and Mucoromycota (class Endogonomycetes)

**DOI:** 10.3897/mycokeys.107.125549

**Published:** 2024-08-09

**Authors:** Leho Tedersoo, Franco Magurno, Saad Alkahtani, Vladimir Mikryukov

**Affiliations:** 1 Mycology and Microbiology Center, University of Tartu, 2 Liivi, 50409 Tartu, Estonia; 2 Department of Zoology, College of Science, King Saud University, 12371 Riyadh, Saudi Arabia; 3 Institute of Biology, Biotechnology and Environmental Protection, Faculty of Natural Sciences, University of Silesia in Katowice, Jagiellońska 28, 40-032 Katowice, Poland; 4 Institute of Ecology and Earth Sciences, University of Tartu, 2 Liivi, 50409 Tartu, Estonia

**Keywords:** Dark taxa, DNA-based classification, holotype, molecular phylogeny, species description

## Abstract

Arbuscular mycorrhizal (AM) fungi - Glomeromycota and Endogonomycetes - comprise multiple species and higher-level taxa that have remained undescribed. We propose a mixed morphology- and DNA-based classification framework to promote taxonomic communication and shed light into the phylogenetic structure of these ecologically essential fungi. Based on eDNA samples and long reads as type materials, we describe 15 new species and corresponding genera (*Pseudoentrophosporakesseensis*, *Hoforsarebekkae*, *Kahvenarebeccae*, *Kelottijaerviashannonae*, *Kungsaengenashadiae*, *Langduoadianae*, *Lehetuaindrekii*, *Lokrumastenii*, *Moosteastephanieae*, *Nikkaluoktamahdiehiae*, *Parniguacraigii*, *Riederbergasylviae*, *Ruuacoralieae*, *Tammsaareavivikae* and *Unemaeeanathalieae*), the genus *Parvocarpum* as well as 19 families (Pseudoentrophosporaceae, Hoforsaceae, Kahvenaceae, Kelottijaerviaceae, Kungsaengenaceae, Langduoaceae, Lehetuaceae, Lokrumaceae, Moosteaceae, Nikkaluoktaceae, Parniguaceae, Riederbergaceae, Ruuaceae, Tammsaareaceae, Unemaeeaceae, Bifigurataceae, Planticonsortiaceae, Jimgerdemanniaceae and Vinositunicaceae) and 17 orders (Hoforsales, Kahvenales, Kelottijaerviales, Kungsaengenales, Langduoales, Lehetuales, Lokrumales, Moosteales, Nikkaluoktales, Parniguales, Riederbergales, Ruuales, Tammsaareales, Unemaeeales, Bifiguratales and Densosporales), and propose six combinations (*Diversisporabareae*, *Diversisporanevadensis*, *Fuscutatacerradensis*, *Fuscutatareticulata*, *Viscosporadeserticola* and *Parvocarpumbadium*) based on phylogenetic evidence. We highlight further knowledge gaps in the phylogenetic structure of AM fungi and propose an alphanumeric coding system for preliminary communication and reference-based eDNA quality-filtering of the remaining undescribed genus- and family-level groups. Using AM fungi as examples, we hope to offer a sound, mixed framework for classification to boost research in the alpha taxonomy of fungi, especially the “dark matter fungi”.

## ﻿Introduction

Arbuscular mycorrhizal (AM) fungi play a crucial role in mineral nutrition and stress alleviation of a vast majority of vascular plants (Brundrett and Tedersoo 2018), especially in the grassland and tropical forest ecosystems (Soudzilovskaia et al. 2019). Besides higher plants, AM fungi also associate with certain liverworts (Marchantiophyta) and hornworts (Anthocerotophyta), forming arbuscule-like structures in their thalli and improving their access to nutrients in soil (Ligrone et al. 2007; Bidartondo and Duckett 2010).

Traditionally, only members of the phylum Glomeromycota (occasionally considered as subphylum Densosporales within Mucoromycota, sensu Spatafora et al. (2016)) have been recognised as AM mycobionts (Smith and Read 2008; Varma et al. 2017). However, there is strong morphological and molecular evidence for AM associations between Endogonomycetes (subphylum Mucoromycotina within Mucoromycota) and various plant groups, including hornworts, liverworts and herbaceous vascular plant species (Bidartondo et al. 2011; Desiro et al. 2013; Bonfante and Venice 2020). Further molecular evidence for endogonomycete associations in plant roots (Orchard et al. 2017b) suggests that these fungi may be as important as Glomeromycota in AM associations in evolutionary (Hoysted et al. 2018) and ecological (Orchard et al. 2017b) terms. Except for *Geosiphon*, taxa of Glomeromycota are recognised as obligate root symbionts, but such information is lacking for Endogonomycetes. Certain small groups of Endogonomycetes are known as ectomycorrhizal symbionts (Tedersoo and Smith 2017) or saprotrophs (Berch and Fortin 1983; Hirose et al. 2014). Members of Endogonomycetes may form macroscopic (semi)hypogeous fruiting bodies containing sexual zygospores or asexual chlamydospores. A few species of Glomeromycota form such fruiting bodies containing chlamydospores (also termed glomerospores), but most species of Glomeromycota produce single glomerospores on hyphal tips in soil. The few *in vitro* culturable species of Glomeromycota can be grown exclusively in co-culture (except *Geosiphon*) with plant roots (but see Tanaka et al. (2022)). Some saprotrophic, ectomycorrhizal and AM species of Endogonomycetes can be grown in pure culture (e.g. Field et al. (2015)).

Given the high attention on Glomeromycota as the primary AM root symbionts, their taxonomy is relatively well established, with three classes, six orders, 17 families and 52 genera accepted (Wijayawardene et al. 2022; Błaszkowski et al. 2022, 2023; da Silva et al. 2023). Conversely, the Endogonomycetes comprise a single order (Endogonales), two families and seven genera (Wijayawardene et al. 2022). The DNA samples of two genera of Endogonomycetes (*Peridiospora* and *Sclerogone*) have never been sequenced due to difficulties accessing old collections.

Much of the Glomeromycota DNA barcoding and phylogenetics research relies on the rRNA internal transcribed spacer (ITS) region and 5’ quarter of the 28S gene (LSU). However, all nuclear LSU, ITS region and 18S rRNA gene (SSU) are nearly equally used for molecular identification from soil and plant roots. The geographically most inclusive studies have focused on either the SSU marker (Davison et al. 2015; Vasar et al. 2022) or the ITS region (Tedersoo et al. 2014, 2021; Kivlin 2020; Mikryukov et al. 2023). Due to the paucity of species-level reference data, the SSU-based surveys suffer relatively more from poor species- and genus-level identification (Tedersoo et al. 2024). In Endogonomycetes, taxonomic studies have used all SSU, ITS and LSU markers. Short-read endogonomycete ITS1 and ITS2 sequences derived from general soil fungal surveys are common in the International Nucleotide Sequence Databases Consortium (INSDC), but the few endogonomycete AM-focused studies have used a long marker fragment of SSU (e.g. Bidartondo et al. (2011); Albornoz et al. (2022)). For the identification from soil or roots, sufficient coverage of both groups requires the use of specific primers. Therefore, focusing on one of the AM groups reduces the amplification of the other (Seeliger et al. 2023). Molecular identification of AM fungi has been heavily biased towards the Glomeromycota, whereas Endogonomycetes have been virtually ignored in 99% of molecular surveys of AM fungi in the last 15 years. For both groups, several undescribed family- or order-level taxa have been revealed based on eDNA, suggesting that much of the taxonomic and phylogenetic diversity remains yet to be described (Desiro et al. 2013; Öpik et al. 2014).

Historically, species of both Endogonomycetes and Glomeromycota have been described in the genus *Endogone* Link (erected by Link (1809)) that was used in a cross-phylum sense until 1980s, although the genus *Glomus* Tul. & C. Tul. was erected for *G.macrocarpum* nearly 150 years earlier (Tulasne and Tulasne 1844). Most of the glomeromycotan species were later transferred to *Glomus* under the family Glomeraceae (Pirozynski and Dalpé 1989), order Glomerales (Morton and Benny 1990), class Glomeromycetes (Cavalier-Smith 1998) and phylum Glomeromycota (Schüssler et al. 2001). In the last two decades, the initial large genera *Glomus* and *Endogone* were split into multiple smaller genera based on combined morphological and molecular analyses. Additional families and orders of Glomeromycota were described by Schüssler et al. (2001), Walker and Schüssler (2004) and Błaszkowski et al. (2021). Gigasporales and Entrophosporales were erected from Glomerales more recently (Gautam and Patel 2007; Błaszkowski et al. 2022). Paraglomerales and Archaeosporales were assigned class rank (Oehl et al. 2011). Recently, the class Endogonomycetes was erected (Doweld 2014) to include the Endogonales (Jaczewski and Jaczewski 1931), covering the mucoromycotan families Endogonaceae (Saccardo 1889) and Densosporaceae (Desiro et al. 2017) as well as two order-level clades “GS21” and “GS22” (Tedersoo et al. 2017) recognised based on soil eDNA samples.

The main purpose of this article is to develop a mixed phylogenetic classification framework that integrates environmental DNA (eDNA) sequences into a specimen-based classification system, which is particularly relevant for high-diversity and cryptic taxonomic groups, such as AM fungi with predicted richness of thousands of species. Already three decades ago, it was stated: “It is unavoidable that DNA will serve as character source for contemporary taxonomic descriptions” (cf. Reynolds and Taylor (1991:311)). Such a mixed morphology- and eDNA-based classification framework is expected to facilitate species discovery and promote work on alpha taxonomy. “Leaving this diversity unnamed or unclassified is not an option, as it would continue to be an enormous and increasing impediment to communication and research in the field” (cf. Lücking and Hawksworth (2018:146)). Fungal species with names improve our capacity to refer to particular organisms and facilitate biodiversity surveys, conservation planning and assessment of toxic, pathogenic and mutualistic organisms in a direct way (Ryberg and Nilsson 2018; Lücking et al. 2021). Furthermore, a well-structured taxonomic hierarchy would offer additional possibilities for using phylodiversity and evolutionary methods without performing phylogenetic analyses (Tedersoo et al. 2018), and it would improve taxonomy-aware chimera filtering in reference-based methods for metabarcoding analyses (Nilsson et al. 2010). The main shortfalls of sequence-based classification include eroding the concept of physical type material and parallel classifications based on specimens and sequences or using different DNA markers (Hongsanan et al. 2018; Lücking and Hawksworth 2018; Thines et al. 2018). Therefore, many leading fungal taxonomists do not approve use of DNA sequences (Thines et al. 2018; Zamora et al. 2018) or eDNA sample (Hongsanan et al. 2018) as holotypes.

Here, we use the mixed specimen-eDNA phylogenetic classification framework to shed light into the phylogenetic diversity of the two groups of AM fungi - Glomeromycota and Endogonomycetes. By using eDNA samples as holotypes (Reynolds and Taylor 1991; Renner 2016), DNA sequences as lectotypes and diagnoses based on molecular differences in ITS and LSU marker genes (Renner 2016), we first describe novel species for the highly divergent groups of AM fungi following the International Code of Nomenclature for Algae, Fungi and Plants (Turland et al. 2018; Lücking et al. 2021). Building on these species, we then introduce novel families and orders. Finally, we provide a large number of taxonomically re-annotated and novel SSU, ITS and LSU sequences, equipped with preliminary alphanumeric taxonomic identifiers, where relevant, to the scientific community.

## ﻿Materials and methods

We downloaded the sequence data identified as Glomeromycota, Mucoromycota and uncultured fungi from three nucleotide sequence databases - NCBI (Sayers et al. 2024; https://www.ncbi.nlm.nih.gov/), UNITE v.9.1 (Abarenkov et al. 2024; https://unite.ut.ee/) and EUKARYOME v.1.7 (Tedersoo et al. 2024; https://eukaryome.org/). We also added rRNA gene sequences from scaffolds in the Joint Genome Institute data portal (https://genome.jgi.doe.gov/portal/). The unidentified fungi were first assigned to rough taxonomic groups based on BLASTn queries against identified sequences in EUKARYOME v.1.7. For sequences affiliated with Glomeromycota or Endogonomycetes, we conducted phylogenetic analyses separately for the SSU, LSU and a longer fragment spanning much of SSU, ITS and LSU. A large part of the ITS region was not used for the phylogeny reconstruction because of alignment unreliability. The sequences of Glomeromycota and Endogonomycetes were aligned using MAFFT v.7 (Katoh and Standley 2013), followed by manual trimming of overarching and misaligned ends and manual correction in case of obvious misalignments using AliView v.1.26 (Larsson 2014). The alignments were further trimmed to exclude unalignable regions and subjected to ClipKIT v.1.4.0 (Steenwyk et al. 2020) to remove phylogenetically uninformative positions, including rare introns and insertions. Based on the alignments, we visually evaluated mismatches to commonly used primers targeting SSU, ITS and LSU regions.

Phylogenetic analyses were performed using IQ-TREE v.2.2.5 (Minh et al. 2020), with standard options including 1000 trees and 1000 ultrafast bootstrap replicates. The trees were visualised and used for taxonomic re-annotation in FigTree v.1.4.4 (Rambaut 2018). Various taxa of Mucoromycota with relatively short branches were tested as potential outgroups. The first three rounds of alignments and analyses were primarily used to detect and remove low-quality reads and chimeric sequences. From the fourth round onwards, the reads were assigned to clades corresponding to putative genera, families and orders, following the monophyly criterion and accounting for the level of sequence divergence in previously described groups. We included at least one read from each described species to delimit clades and assign taxonomy. For both Glomeromycota and Endogonomycetes, we focused mainly on the long fragment covering the ITS and LSU regions because of: 1) the greatest taxonomic resolution in the ITS2 and D2 subregion of LSU, 2) the occurrence of the largest number of described species and 3) the presence of most abundant and diverse set of eDNA reads from soil and roots falling into these groups (Tedersoo et al. 2024).

Diagnoses of species were prepared based on molecular characters in the ITS and LSU regions by selecting the most characteristic short barcodes (20–30 bases) for the target species using multiple sequence alignments. The barcodes typically had no ambiguous position for the target species and had at least two differences from closely-related species. We also estimated the number of mutations (i.e. alignment mismatches) allowed for the target species to be separable from related species (typically set to 0 or 1). For the entire alignment length of ITS and LSU, we estimated the maximum proportion of differences amongst sequences corresponding to the target species (i.e. within-species variability).

For establishing higher-ranking taxa such as genera, families and orders, we used the following criteria: i) monophyly; ii) bootstrap support >95; iii) phylogenetic breadth and divergence roughly comparable to previously described taxa; and iv) minimising the number of novel taxa (i.e. preferably retaining larger groups if there were multiple alternative splitting possibilities). Based on a visual assessment of the ITS and LSU alignments and phylograms, we predicted the approximate number of (potential) species for each newly-described genus (but extrapolation to unobserved taxa was not attempted).

The eDNA samples with the highest proportions of target reads were selected as holotypes, except in the cases where long reads spanning SSU, ITS and LSU were available along with the stored DNA samples. Lectotypes were identified amongst the highest-quality sequences derived from these holotype DNA samples. Most of the type materials and additional samples were derived from composite topsoil samples (40 subsamples of 5 cm diam. to 5 cm depth from 2500-m^2^ area) of the Global Soil Mycobiome consortium (GSMc) project (Tedersoo et al. 2021), FunAqua sediment samples (V. Prins et al., unpublished) or from various soil samples sequenced by Jamy et al. (2022). Both eDNA and corresponding substrate samples are maintained as vouchered collections in the repository of the University of Tartu (acronym TUE, with 6-digit accession numbers). The sequences were first deposited in the EUKARYOME database (denoted by “EUK” with 7-digit accession numbers) and subsequently submitted to the INSDC and UNITE databases. EUKARYOME v.1.9.2 includes 55,648 and 10,081 annotated reads of Glomeromycota and Endogonomycetes, respectively.

## ﻿Results

### ﻿Phylogeny of Glomeromycota

**Figure 1.**
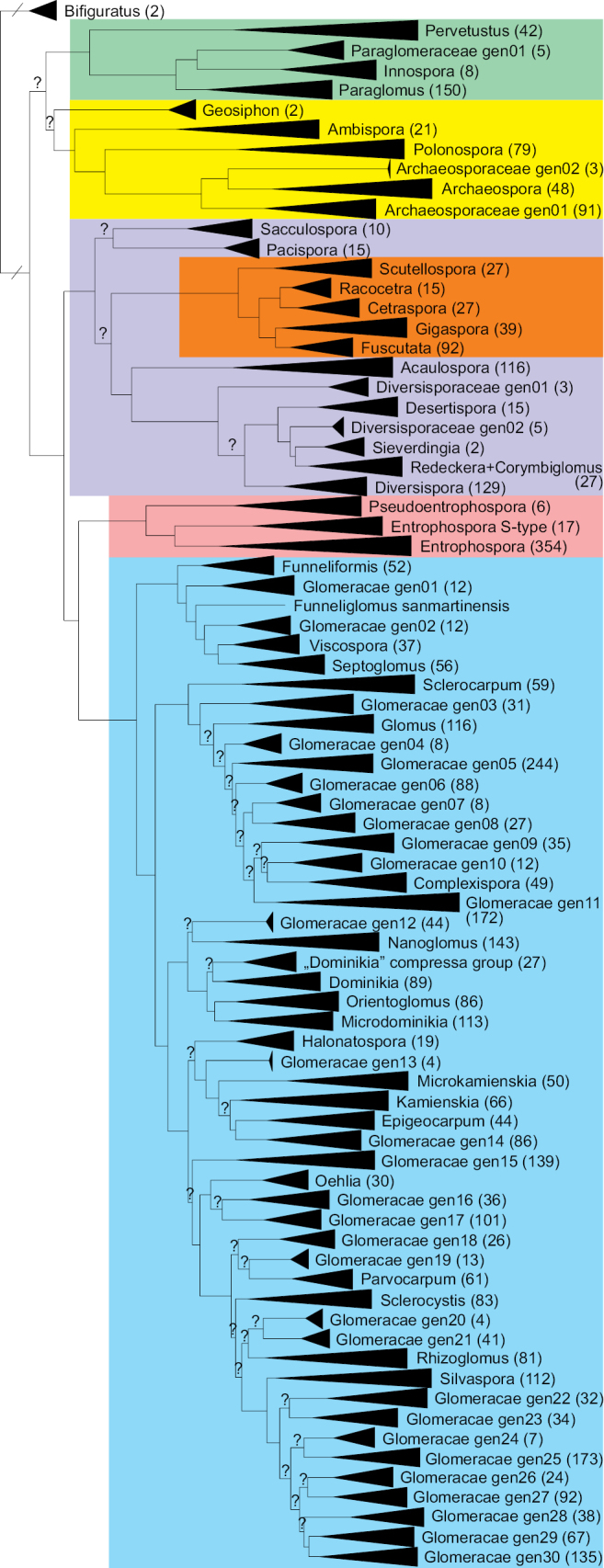
Phylogenetic position of genera and genus-level groups of Glomeromycota based on Maximum Likelihood analysis of SSU-ITS-LSU sequences. The clades are collapsed, with the number of sequences included in parentheses. Question marks above branches indicate low ultra-rapid bootstrap support (< 95). Orders are highlighted in different colours. For the genus *Entrophospora*, the so-called S-type reads (VanKuren et al. 2013) were included for comparison. The uncollapsed phylogram is given in Suppl. material 1.

The SSU-ITS-LSU phylogram supported the separation of all described Glomeromycota orders and families, and placed these into expected positions (Fig. 1, Suppl. material 1) as in previous analyses based on rRNA gene and partial genomes (Stockinger et al. 2012; Montoliu-Nerin et al. 2021; Rosling et al. 2024). A vast majority of valid genera were separated from each other with high statistical support. As exceptions, the genera *Otospora* Oehl, Palenzuela & N.Ferrol (*O.bareae*) and *Tricispora* Oehl, Sieverd., G.A.Silva & Palenz. (*T.nevadensis*) were nested within *Diversispora* C.Walker & A.Schüssler, whereas species of *Dentiscutata* Sieverd., F.A.Souza & Oehl (*D.cerradensis* and *D.reticulata*) were placed within *Fuscutata* Oehl, F.A.Souza & Sieverd. The type species of *Dentiscutata* (*D.nigra*) was not sequenced for the ITS region, but an analysis of the LSU region indicated that *D.nigra* is placed separately from other species of *Dentiscutata* that clustered with *Fuscutata* (Suppl. material 2). *Corymbiglomuscorymbiforme* Błaszk. & Chwat – the type species of this genus – was nested within the genus *Redeckera* C.Walker & A.Schüssler, whereas *C.globiferum* (Koske & C.Walker) Błaszk. & Chwat served as a sister group to species of *Redeckera*. Furthermore, the recently described genus *Blaszkowskia* G.A.Silva & Oehl was nested within *Viscospora*. Where relevant, we propose new combinations (see below).

Of previously described species, *Dominikiacompressa* (Sieverd., Oehl, Palenz., Sánchez-Castro & G.A.Silva) Oehl, Palenz., Sánchez-Castro & G.A.Silva (basionym *Glomuscompressum* Sieverd., Oehl, Palenz., Sánchez-Castro & G.A.Silva) formed a well-supported group in a sister position to the rest of *Dominikia* Błaszk., Chwat & Kovács, but their close relationship was poorly supported and inconsistent amongst various phylograms prepared. Thus, *D.compressa* is currently being transferred to a new genus (J. Błaszkowski et al., in prep.). Similarly, *Glomusbadium* Oehl, D.Redecker & Sieverd. was placed outside the genus *Glomus* as a well-supported small clade, but its sister relationships with other genera remain unresolved. Based on both phylogenetic and morphological characters, we propose to treat *G.badium* as a new genus, herein designated as *Parvocarpum* (see below).

Our phylogenetic analysis revealed a large number of previously undescribed or unsequenced taxa. One of these taxa was located as a deep clade in the Entrophosporales, which warrants consideration as a new family outside the Entrophosporaceae. We describe the new species, genera and families based on eDNA samples and sequences. The Archaeosporaceae and Diversisporaceae families each revealed two novel genus-level taxa, whereas the Paraglomeraceae harboured one new genus-level taxon. The most prominent family – Glomeraceae – was found to include 30 novel genus-level taxa clearly distinct from current delimitations of known genera based on our criteria. We propose informal alphanumeric labels for these genera to enable their communication (see below). For the Glomeraceae, it is most likely that, upon DNA sequencing of materials belonging to unsequenced species, many will fall into these unnamed groups (like the cases of *D.compressa* and *G.badium*).

### ﻿Phylogeny of Endogonomycetes

**Figure 2.**
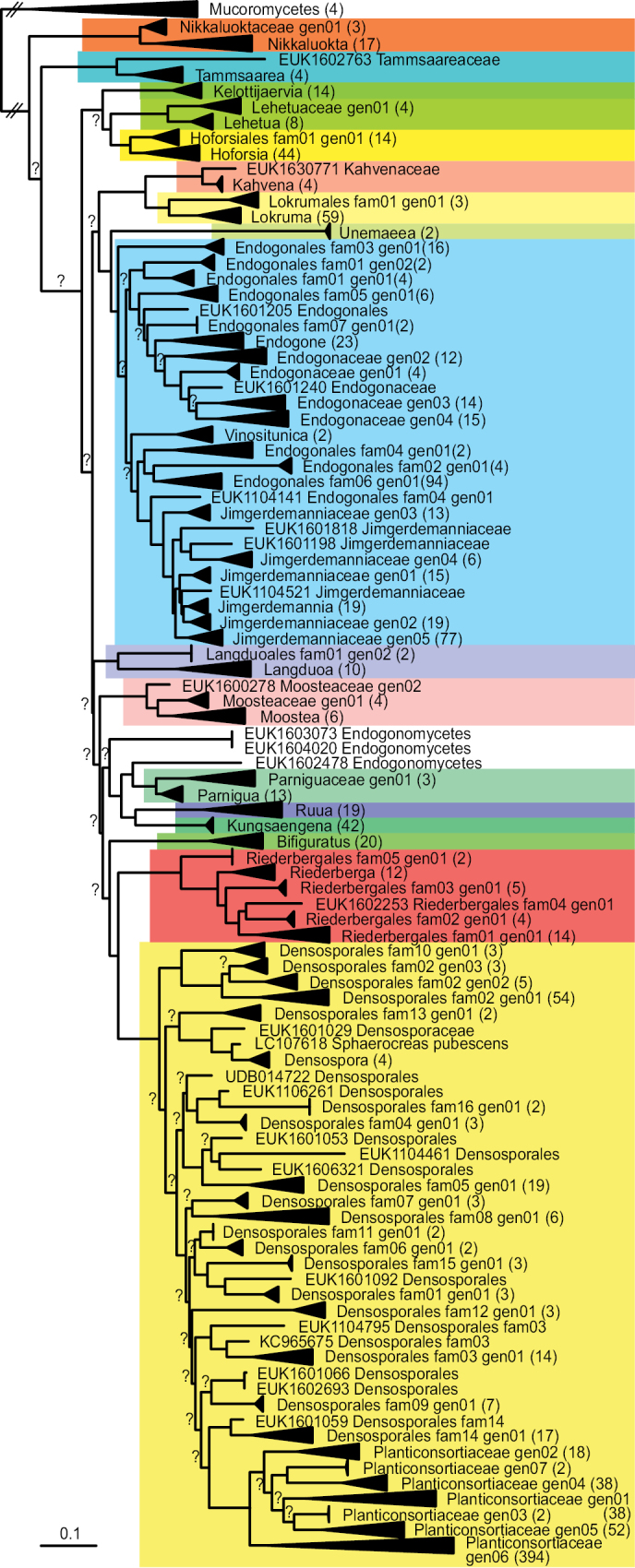
Phylogenetic position of genera and genus-level groups of Endogonomycetes based on Maximum Likelihood analysis of SSU-5.8S-LSU sequences. The clades are collapsed, with the number of sequences included in parentheses. Question marks above branches indicate low ultra-rapid bootstrap support (< 95). Orders are highlighted in different colours. The uncollapsed phylogram is given in Suppl. material 3.

The SSU-5.8S-LSU phylogram resolved the internal structure of Endogonomycetes reasonably well, except the order of divergence for most of the 17 main, deep-branching groups (Fig. 2, Suppl. material 3). The hitherto described and sequenced species of *Endogone* and *Jimgerdemannia* Trappe, Desirò, M.E.Sm., Bonito & Bidartondo, as well as *Densospora* McGee and *Sphaerocreas* Sacc. & Ellis, formed two relatively large order-level groups that were distantly related to each other and surrounded by eDNA sequences derived from soil. Species of *Bifiguratus* Torres-Cruz & Porras-Alfaro formed a small, deep-branching, order-level group, with no clear sister group. For each of the additional 14 order-level groups, we described new species based on eDNA sample and sequence information. These 14 species were further assigned to genera and families based on the internal branching structure of these orders, with other unnamed groups labelled alphanumerically. Two potentially order-level groups remain undescribed and unlabelled, because these were found from a single locality and their phylogenetic position may change with extra sequences and more precise alignments.

The additional SSU phylogram confirmed the separation of the main orders, although nearly half of them lacked SSU sequence data or were represented by a single read (Suppl. material 4). Furthermore, the SSU phylogram indicated that the vast majority of putative AM fungi were widely dispersed in the groups corresponding to Densosporales (mostly Densosporaceae and Planticonsortiaceae), Endogonales (including Endogonaceae, Jimgerdemanniaceae and other family-level taxa) and Hoforsales (formerly clade GS22), and to some extent in Bifiguratales. However, a few root-derived sequences fell outside these groups, suggesting that certain other orders lacking the SSU sequence data may also host AMF.

### ﻿Taxonomic combinations, emendations and descriptions in Glomeromycota

#### ﻿ Diversispora bareae

Taxon classificationFungiDiversisporalesDiversisporaceae

(Palenz., N.Ferrol & Oehl) Tedersoo & Magurno comb. nov.

DA57AE55-27B2-5141-BD8F-039113A0E4D3

853545

Otospora bareae Palenz., N.Ferrol & Oehl, in Palenzuela, Ferrol, Boller, Azcón-Aguilar & Oehl, Mycologia 100(2): 298 (2008). Basionym.

##### Description.

As presented originally in Palenzuela et al. (2008).

##### Diagnosis.

*Diversisporabareae* differs from other species of the *Diversispora* by producing acaulosporoid (otosporoid) spores compared with diversisporoid and entrophosporoid (tricisporoid) spores in other described species. Glomerospores with inner flexible hyaline layer and pigmented sporiferous saccule. Phylogenetically belongs to *Diversispora* based on the SSU-ITS-LSU phylogram (Fig. 1, Suppl. material 1).

##### Notes.

The new combination invites an amendment of the genus *Diversispora* to accommodate species with otosporoid spores.

#### ﻿ Diversispora nevadensis

Taxon classificationFungiDiversisporalesDiversisporaceae

(Palenz., N.Ferrol, Azcón-Aguilar & Oehl) Tedersoo & Magurno comb. nov.

64AF15C7-631D-5EF7-B44C-D219A627DB40

853546

Entrophospora nevadensis Palenz., N.Ferrol, Azcón-Aguilar & Oehl, in Palenzuela, Barea, Ferrol & Azcón-Aguilar, Mycologia 102(3): 627 (2010). Basionym.

##### Description.

See Palenzuela et al. (2010).

##### Diagnosis.

*Diversisporanevadensis* differs from other species of the *Diversispora* by producing entrophosporoid (tricisporoid) spores compared with diversisporoid and acaulosporoid (otosporoid) spores in other species. Glomerospores with inner flexible hyaline wall layers without granular beaded surface and no Melzer reaction. Phylogenetically nested in *Diversispora* based on the SSU-ITS-LSU phylogram (Fig. 1, Suppl. material 1).

##### Notes.

The new combination invites an amendment of the genus *Diversispora* to accommodate species with entrophosporoid (tricisporoid) spores.

#### ﻿ Diversispora

Taxon classificationFungiDiversisporalesDiversisporaceae

C.Walker & A.Schüssler emend. Tedersoo & Magurno

1613A7B6-E4AE-55A3-A34A-9797979835A7

28884

##### Type species.

*Diversisporaspurca* (C.M.Pfeiffer, C.Walker & Bloss) C.Walker & Schüssler.

##### Description.

Spores diversisporoid, rarely otosporoid or tricisporoid. Diversisporoid spores formed singly, in clusters or in large disorganised fruiting bodies with high spore numbers. Spores with 1–4 wall layers; pores often closed with a septum. Subtending hyphal pores rarely open. Otosporoid spores formed laterally on the persistent neck of a sporiferous saccule. Tricisporoid spores with inner flexible hyaline wall layers (formed de novo) without granular beaded surface and no Melzer reaction. Spore pores generally closed by a septum at the spore base, arising from the innermost wall lamina or inner layer or from both. Forms a monophyletic group within Diversisporaceae based on the SSU-ITS-LSU phylogram (Fig. 1, Suppl. material 1).

#### ﻿ Fuscutata cerradensis

Taxon classificationFungiDiversisporalesGigasporaceae

(Spain & J. Miranda) Tedersoo & Magurno comb. nov.

C73638C5-35FF-5D57-8DD7-7EAC3C32C667

853547

Scutellospora cerradensis Spain & J. Miranda, Mycotaxon 60: 130 (1996). Basionym.
Dentiscutata cerradensis Sieverd., F.A.Souza & Oehl, Mycotaxon 106: 342 (2009). Synonym.

##### Description.

See Spain and Miranda (1996).

##### Diagnosis.

*Fuscutatacerradensis* differs from other species of the *Fuscutata* by spore wall ornamentation, three-walled spores and dark-pigmented multilobed germinal shield produced in the inner wall. Phylogenetically forms a monophyletic clade with *F.heterogama* - the type species of genus - based on the SSU-ITS-LSU phylogram (Fig. 1, Suppl. material 1).

##### Notes.

The new combination invites an amendment of genus *Fuscutata* to accommodate species with dark, multilobed germinal shields. However, we decided not to prepare an amendment for *Fuscutata* because the genus *Dentiscutata*, their close relative, requires additional information to confirm their status, supported only in the LSU sequence of *D.nigra*.

#### ﻿ Fuscutata reticulata

Taxon classificationFungiDiversisporalesGigasporaceae

(Koske, D.D.Mill. & C.Walker) Tedersoo & Magurno comb. nov.

BBD6E7DB-B766-5D21-BBAE-BB0CC7E23B0A

853548

Gigaspora reticulata Koske, D.D.Mill. & C.Walker, Mycotaxon 16(2): 429 (1983). Basionym.
Dentiscutata reticulata (Koske, D.D.Mill. & C.Walker) Sieverd., F.A.Souza & Oehl, Mycotaxon 106: 342 (2009). Synonym.

##### Description.

See Koske et al. (1983).

##### Diagnosis.

*Fuscutatareticulata* differs from other species of the *Fuscutata* by spore wall ornamentation, three-walled spores and dark-pigmented, multilobed germinal shield produced in the inner wall. Phylogenetically forms a monophyletic clade with *F.heterogama* - type species of genus - based on the SSU-ITS-LSU phylogram (Fig. 1, Suppl. material 1).

##### Notes.

See note of *F.cerradensis*.

#### ﻿ Viscospora deserticola

Taxon classificationFungiGlomeralesGlomeraceae

(Trappe, Bloss & J.A.Menge) Tedersoo & Magurno comb. nov.

40E147F5-E4AD-5218-9307-DE6B082407FB

853549

Glomus deserticola Trappe, Bloss & J.A.Menge, Mycotaxon 20 (1): 123 (1984). Basionym.
Blaszkowskia deserticola (Trappe, Bloss & J.A.Menge) Oehl & G.A.Silva, Mycol. Progr. 22 (11, no. 74): 5 (2023). Synonym.

##### Description.

See Trappe et al. (1984).

##### Diagnosis.

Subtending hyphae pigmented over long distances (>100 μm) unlike in other species of *Viscospora* and *Septoglomus*. Differs from other species of *Viscospora* by spore colour (da Silva et al. 2023).

##### Notes.

Transferred from *Blaszkowskia* to *Viscospora* because of phylogenetic nestedness within *Viscospora* and recognition as a separate genus would render *Viscospora* paraphyletic and leave many orphan taxa in the *Septoglomus*-*Viscospora* clade (Suppl. material 1; da Silva et al. (2023)).

#### ﻿ Parvocarpum

Taxon classificationFungiGlomeralesGlomeraceae

Magurno gen. nov.

EAB06364-E6B2-57A0-BADE-E28C34CA886E

853558

##### Type species.

*Parvocarpumbadium* (Oehl, Redecker & Sieverd.) Magurno.

##### Description.

Producing glomoid-like spores surrounding a central plexus of interwoven hyphae in small organised fruiting bodies, lacking a peridium. Spores with inner flexible hyaline layer and short subtending hyphae. Forms a monophyletic group within Glomeraceae based on SSU-ITS-LSU phylogram (Fig. 1, Suppl. material 1).

##### Notes.

Based on ITS and LSU sequences, *Parvocarpum* includes 10–20 species.

#### ﻿ Parvocarpum badium

Taxon classificationFungiGlomeralesGlomeraceae

(Oehl, Redecker & Sieverd.) Magurno comb. nov.

35B241B1-E9A2-51F5-B40C-EE7943B4FEDF

853560

Glomus badium Oehl, D.Redecker & Sieverd., Angew. Botan. 79: 39 (2005). Basionym.
Funneliformis badius (Oehl, Redecker & Sieverd.) C.Walker & A.Schüssler. Synonymy.\

##### Description.

See Oehl et al. (2005).

##### Etymology.

*parvus* (Latin) = small; and *carpum* (Latin) = body, referring to the small size of fruiting bodies produced.

##### Diagnosis.

*P.badium* differs from other genera of the Glomeraceae by producing glomoid-like spores surrounding a central plexus of interwoven hyphae in small organised fruiting bodies, lacking a peridium. Spores with inner flexible hyaline layer and short subtending hyphae. Phylogenetically distinct from *G.macrocarpum* and other *Glomussens. str.* species based on the SSU-ITS-LSU phylogram (Fig. 1, Suppl. material 1).

##### Notes.

Phylogenetic position of *P.badium* within the genus *Parvocarpum* is unresolved because of a single available short read.

#### ﻿ Pseudoentrophosporaceae

Taxon classificationFungiEntrophosporales

Tedersoo & Magurno fam. nov.

439F61CA-62E7-587A-925F-F81FC46C1DBE

853564

##### Type genus.

*Pseudoentrophospora* Tedersoo & Magurno.

##### Description.

Covers the monophyletic group in Entrophosporales (Fig. 1). Phylogenetically delimited as the least inclusive clade covering sequence accessions EUK1631429, EUK1105140 and EUK0135500 (Suppl. material 1).

##### Notes.

Recognised based on eDNA sequences only. Currently monogeneric.

#### ﻿ Pseudoentrophospora

Taxon classificationFungiEntrophosporales﻿Pseudoentrophosporaceae

Tedersoo & Magurno gen. nov.

799AECC1-5536-50BB-8D37-1A618B2DA4F8

853565

##### Type species.

*Pseudoentrophosporakesseensis* Tedersoo & Magurno.

##### Description.

Covers the monophyletic group in Pseudoentrophosporaceae (Fig. 1). Phylogenetically delimited as the least inclusive clade covering sequence accessions EUK1631429, EUK1105140 and EUK0135500 (Suppl. material 1).

##### Notes.

Recognised based on eDNA sequences only. There are potentially 3–6 species in *Pseudoentrophospora* based on ITS sequences, some of which are represented by sequences EUK1105140 (tropical rainforest soil in El Yunque, Puerto Rico, 18.29°N, -65.78°E); EUK1010525 (GSMc plot S056, tropical rainforest soil in Pegaima Mountains, Guyana, 5.43567°N, -60.08825°E); and EUK0133825 (flooded grassland soil in Dijle, Belgium, 5.83°N, 4.65°E).

#### ﻿ Pseudoentrophospora kesseensis

Taxon classificationFungiEntrophosporales﻿Pseudoentrophosporaceae

Tedersoo & Magurno sp. nov.

3EF89F07-3237-56C3-AB4B-37AEDC4F9360

853566

##### Diagnosis.

**Figure 3.**
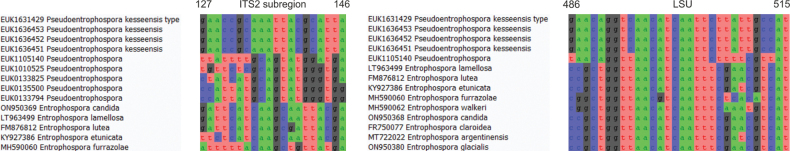
Diagnostic barcodes for *Pseudoentrophosporakesseensis* relative to closely-related taxa in ITS2 and LSU.

Differs from other species of *Pseudoentrophospora* and *Entrophospora* based on the ITS region (ITS2 positions 127–146 gaaccgcaaattacgcatta, one mismatch allowed) and LSU (positions 486–515 gaacaggtcaacatcaattcttattgccat, one mismatch allowed) as indicated in Fig. 3.

##### Type.

Soil eDNA sample TUE101916 (***holotype***); eDNA sequence EUK1631429 (***lectotype***); GSMc plot G4940, coppiced *Juniperus*-*Acer* woodland (soil sample TUE001916) in Kesse Island, Estonia, 58.63443°N, 23.43938°E.

##### Description.

Other eDNA sequences EUK1636430–EUK1636432 from the type locality.

##### Etymology.

*pseudo* (Greek) = false; *Entrophospora* (Latin) refers to a related fungal genus; and *kesseensis* (Latin) indicates locality of the type species. The name depicts phylogenetic relatedness to *Entrophosphora* and the only locality where the type species has been recorded.

##### Notes.

Found from a single site, with ITS and LSU sequences differing up to 0.5% and 1%, respectively. The ITS1 subregion harbours only 58 bases, being amongst the shortest across fungi (excl. microsporidians).

### ﻿Taxonomic descriptions of Endogonomycetes

#### ﻿ Endogonomycetes

Taxon classificationFungiEndogonomycetes

Doweld emend. Tedersoo

2E5A33C3-7C77-5BC3-8FE8-3A22AA0ADEB6

550357

##### Type order.

Endogonales Jacz. & P.A.Jacz.

##### Description.

Fruiting body absent, rarely present - hypogeous or on debris, globose, irregular, sometimes resupinate, 1–20 mm in diam., may be composed of aggregated zygosporangial clusters. Reproductive structures as zygosporangia (in *Endogone*, *Jimgerdemannia*) or chlamydospores (in *Vinositunica*, *Densospora*), aggregated in the fruiting body or as chlamydospores on extraradical hyphae (in *Planticonsortium*). Chlamydospore wall continuous, multilayered, with dense subtending hyphae, lacking septa. Hyphae filamentous, coenocytic, sometimes with secondary septa, rarely yeast-like (in *Bifiguratus*). Forms a monophyletic group in Mucoromycota, as the least inclusive clade covering accessions UDB025468, UDB28692, EUK1201418, EUK1203196, EUK1602762, EUK1202520, EUK1203766, EUK1107335 and EUK1602357 (Suppl. material 3).

##### Notes.

Endogonomycetes harbours currently 17 orders and two unassigned, potentially order-level groups represented by sequences EUK1604020 and EUK1603073 (GSMc plot G3308, *Juniperuscommunis* coppiced grassland soil in Atla, Estonia, 58.30122°N, 21.93600°E); and EUK1602478 (GSMc plot G4627, mixed forest soil in Tudusoo, Estonia, 59.11368°N, 26.75944°E).

#### ﻿ Hoforsales

Taxon classificationFungi

Tedersoo ord. nov.

BE12D337-D710-57AD-9C3D-BA3EBCFB80C5

853567

##### Type family.

Hoforsaceae Tedersoo.

##### Description.

Covers the monophyletic group in Endogonomycetes (Fig. 2). Phylogenetically delimited as the least inclusive clade covering sequence accessions EUK1100001, EUK1602331 and EUK1602346 (Suppl. material 3).

##### Notes.

Recognised based on eDNA sequences only. Currently includes Hoforsaceae and another potentially family-level group, which is represented by sequence EUK1631675 (GSMc plot G4124, *Populustremula* forest soil in Mäla, Estonia, 58.58693°N, 23.28597°E). Hoforsales corresponds to clade GS22 (sensu Tedersoo et al. (2017)).

#### ﻿ Hoforsaceae

Taxon classificationFungiHoforsales

Tedersoo fam. nov.

F545EB4C-FA9F-5CAC-A79E-85C9BAF058D5

853569

##### Type genus.

*Hoforsa* Tedersoo.

##### Description.

Covers the monophyletic group in Hoforsales (Fig. 2). Phylogenetically delimited as the least inclusive clade covering sequence accessions EUK1100001, EUK1107311 and EUK1602325 (Suppl. material 3).

##### Notes.

Recognised based on eDNA sequences only. Currently monogeneric.

#### ﻿ Hoforsa

Taxon classificationFungiHoforsalesHoforsales

Tedersoo gen. nov.

80B9FA9A-DD5D-56D0-87AE-A603667661BB

853570

##### Type species.

*Hoforsarebekkae* Tedersoo.

##### Description.

Covers the monophyletic group in Hoforsaceae (Fig. 2). Phylogenetically delimited as the least inclusive clade covering sequence accessions EUK1100001, EUK1107311 and EUK1602325 (Suppl. material 3).

##### Notes.

Recognised based on eDNA sequences only. There are potentially about 20 species in *Hoforsa* based on ITS and LSU sequences, with examples including taxa represented by sequences EUK1107311 (bog peat in Svartberget, Sweden, 64.24°N, 19.76°E) and AM260926 (bog peat, Scotland) first isolated by Rebekka Artz (Artz et al. 2007). Most taxa are found from various soils, but the LSU sequence AB982123 originates from an ectomycorrhizal root of Dipterocarpaceae (Lambir, Malaysia). The most common taxon at 99% LSU sequence similarity (EUK1602281) has been recorded from 31 localities in Estonia and Latvia. The genus has a global distribution and it occurs commonly in soil samples but rarely in roots.

#### ﻿ Hoforsa rebekkae

Taxon classificationFungiHoforsalesHoforsaceae

Tedersoo sp. nov.

2025C719-6EB2-5F88-82D3-678705158197

853571

##### Diagnosis.

**Figure 4.**
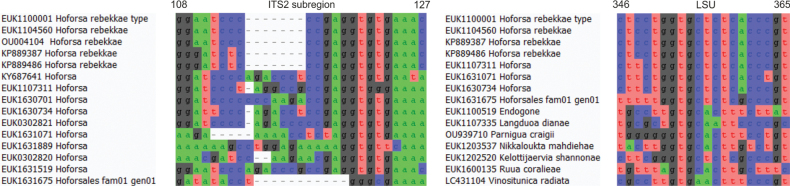
Diagnostic barcodes for *Hoforsarebekkae* relative to closely-related taxa in ITS2 and LSU.

Separation from other species of *Hoforsa* based on the ITS region (ITS2 positions 108–127 ggratcycccgaggtgtgaaac; one mismatch allowed) and LSU (positions 546–565 ctcctggtgctctcacccgt; no mismatch allowed) as indicated in Fig. 4.

##### Type.

Soil eDNA sample: TUE128830 (***holotype***); eDNA sequence EUK1100001 (***lectotype***); *Pinussylvestris* forest near Hofors, Sweden (60.49°N, 16.30°E).

##### Description.

Other sequences: EUK1104560 (type locality); OU004104 (San Francisco, Ecuador, root sample); and KP889387 and KP889486 (both coniferous forest soil in British Columbia, Canada).

##### Etymology.

*Hofors* (Swedish) refers to type locality; and *Rebekka* (Scotch) refers to the first name Rebekka Artz who was the first to collect materials from this genus.

##### Notes.

Found from three sites across three continents, with ITS sequences differing up to 3.5% and LSU sequences up to 0.5%.

#### ﻿ Kahvenales

Taxon classificationFungi

Tedersoo ord. nov.

00199A53-C7D8-5E01-9356-0081EBCF84FA

853572

##### Type family.

Kahvenaceae Tedersoo.

##### Description.

Covers the monophyletic group in Endogonomycetes (Fig. 2). Phylogenetically delimited as the least inclusive clade covering sequence accessions EUK1634339 and EUK1630771 (Suppl. material 3).

##### Notes.

Recognised based on eDNA sequences only. Currently includes Kahvenaceae.

#### ﻿ Kahvenaceae

Taxon classificationFungiKahvenales

Tedersoo fam. nov.

8DEBCDA3-A185-54F9-BBA8-F5B2295633EC

853573

##### Type genus.

*Kahvena* Tedersoo.

##### Description.

Covers the monophyletic group in Kahvenales (Fig. 2). Phylogenetically delimited as the least inclusive clade covering sequence accessions EUK1634339 and EUK1630771 (Suppl. material 3).

##### Notes.

Recognised based on eDNA sequences only. Currently includes *Kahvena*.

#### ﻿ Kahvena

Taxon classificationFungiKahvenalesKahvenaceae

Tedersoo gen. nov.

F24FD4C7-2094-5DC7-A432-F2D97A5626EB

853574

##### Type species.

*Kahvenarebeccae* Tedersoo.

##### Description.

Covers the monophyletic group in Kahvenaceae (Fig. 2). Phylogenetically delimited as the least inclusive clade covering sequence accessions EUK1634339 and EUK1630771 (Suppl. material 3).

##### Notes.

Recognised based on eDNA sequences only. Based on ITS sequences, *Kahvena* is comprised of two species; the other represented by sequences EUK1630771 (GSMc plot G4185, *Picea-Pinus* forest soil in Ristipalo, Estonia, 58.10241°N, 27.47874°E) and ON963629 (*Pinussylvestris* forest soil, Lithuania).

#### ﻿ Kahvena rebeccae

Taxon classificationFungiKahvenalesKahvenaceae

Tedersoo sp. nov.

EB20E4EE-572F-53DB-849A-4606F20139EB

853575

##### Diagnosis.

**Figure 5.**
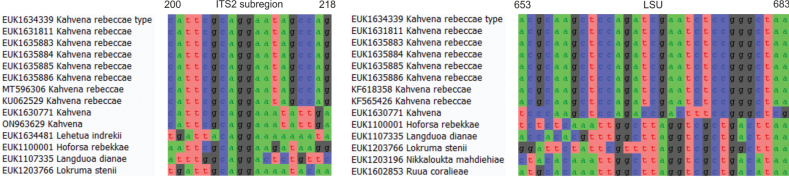
Diagnostic barcodes for *Kahvenarebeccae* relative to closely-related taxa in ITS2 and LSU.

Separation from other species of *Kahvena* based on the ITS region (ITS2 positions 200–218 cattcgcaggaatagccag; one mismatch allowed) and from other species of Endogonomycetes based on LSU (positions 653–683 acgcaagctccagatcgaatctccgggctaa; one mismatch allowed) as indicated in Fig. 5.

##### Type.

Soil eDNA sample TUE100738 (***holotype***); eDNA sequence EUK1634339 (***lectotype***); GSMc plot G4196, *Populus-Picea-Pinus* forest (soil sample TUE000738) in Kahvena, Estonia (58.27991°N, 25.23165°E).

##### Description.

Other sequences: EUK1635883–EUK1635886 (type locality); EUK1631811 (GSMc plot G2767, mixed woodland soil at Mäebe, Estonia, 58.30937°N, 22.07618°E); KF618358 (*Piceamariana* forest soil, AK, USA); MT596306 (Tobiotsuka Kofun, Japan, 34.6355°N, 133.6814°E); KU062529 (unknown source); and KF565426 (Duke Forest, NC, USA, 35.97°N, -79.09°E), isolated by Rebecca C. Mueller (Mueller et al. 2014).

##### Etymology.

*Kahvena* (Estonian) refers to type locality; and *Rebecca* (English) refers to the first name of Rebecca C. Mueller, who collected the first materials belonging to this genus and the type species.

##### Notes.

Found from temperate and subarctic forests in Europe, Asia and North America, with ITS and LSU sequences differing up to 4% (excluding a 29-base deletion in EUK1631811 and KU062529) and 1.5%, respectively. Considered as a single species because of high intraspecific variation amongst common sequence variants in the type locality (2% in ITS and 1% in LSU, representing both indels and substitutions).

#### ﻿ Kelottijaerviales

Taxon classificationFungi

Tedersoo ord. nov.

D66B982B-4882-54A1-B6F9-D49D9073B6CD

853576

##### Type family.

Kelottijaerviaceae Tedersoo.

##### Description.

Covers the monophyletic group in Endogonomycetes (Fig. 2). Phylogenetically delimited as the least inclusive clade covering sequence accessions EUK1202520 and EUK1633699 (Suppl. material 3).

##### Notes.

Recognised based on eDNA sequences only. Currently includes Kelottijaerviaceae.

#### ﻿ Kelottijaerviaceae

Taxon classificationFungiKelottijaerviales

Tedersoo fam. nov.

BBC931E5-D7C2-51AF-8556-A9DB1B4080D9

853577

##### Type genus.

*Kelottijaervia* Tedersoo.

##### Description.

Covers the monophyletic group in Kelottijaerviales (Fig. 2). Phylogenetically delimited as the least inclusive clade covering sequence accessions EUK1202520 and EUK1633699 (Suppl. material 3).

##### Notes.

Recognised based on eDNA sequences only. Currently includes *Kelottijaervia*.

#### ﻿ Kelottijaervia

Taxon classificationFungiKelottijaervialesKelottijaerviaceae

Tedersoo gen. nov.

B94EFDDD-356D-5FE4-A92E-A2C0ACB93E7A

853578

##### Type species.

*Kelottijaerviashannonae* Tedersoo.

##### Description.

Covers the monophyletic group in Kelottijaerviaceae (Fig. 2). Phylogenetically delimited as the least inclusive clade covering sequence accessions EUK1202520 and EUK1633699 (Suppl. material 3).

##### Notes.

Based on ITS and LSU sequences, *Kelottijaervia* is comprised of about five species that are represented by sequences EUK1603128 (GSMc plot G2755X, *Pinussylvestris* forest soil, Liiva-Putla, Estonia, 58.38859°N, 22.65545°E); EUK0302816 (plot G5403, mixed coniferous forest in Kõrveküla, Estonia, 58.43789°N, 26.75099°E); EUK1104755 (*Pinussylvestris* forest soil near Hofors, Sweden, 60.49°N, 16.30°E); and KP889573 (coniferous forest soil in British Columbia, Canada). The genus seems to prefer acidic coniferous forest habitats.

#### ﻿ Kelottijaervia shannonae

Taxon classificationFungiKelottijaervialesKelottijaerviaceae

Tedersoo sp. nov.

E3587C65-1087-50AE-BCB0-CA7ADC90CAE1

853579

##### Diagnosis.

**Figure 6.**
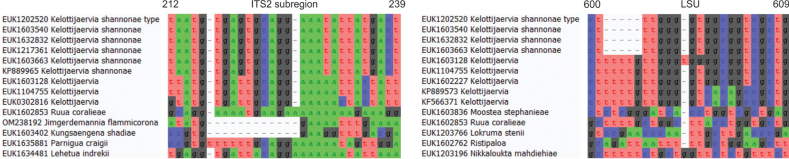
Diagnostic barcodes for *Kelottijaerviashannonae* relative to closely-related taxa in ITS2 and LSU.

Separation from other species of *Kelottijaervia* based on the ITS region (positions 212–239 taatgtgagtgcaggaaatattatgact; one mismatch allowed) and LSU (positions 600–619 ctttggggtggcggtcgctg; one mismatch allowed) as indicated in Fig. 6.

##### Type.

eDNA sample TUE100189 (***holotype***); eDNA sequence EUK1202520 (***lectotype***); GSMc plot G2836 Finland, subpolar *Betulapubescens* forest (soil sample TUE000189) in Kelottijärvi, Finland, 68.60353°N, 21.74517°E.

##### Description.

Other sequences: EUK1603540, (GSMc plot G4196, *Populus-Picea-Pinus* forest soil in Kahvena, Estonia, 58.27991°N, 25.23165°E); EUK1603663 (GSMc plot G4406, mixed coniferous forest soil in Tarumaa, Estonia, 59.20745°N, 27.15333°E); EUK1602832 (GSMc plot G5828, *Malusdomestica* orchard soil in Mooste, Estonia, 58.15335°N, 27.19642°E); and KP889965 (coniferous forest soil in British Columbia, Canada) that was first isolated by Shannon H.A. Guichon (Guichon 2015).

##### Etymology.

*Kelottijärvi* (Finnish) refers to type locality; and *Shannon* (English) refers to the first name of Shannon H.A. Guichon who collected the first materials belonging to this genus.

##### Notes.

Found in Estonia, Finland and Canada, with ITS and LSU sequences displaying up to 2% and 1% of differences, respectively.

#### ﻿ Kungsaengenales

Taxon classificationFungi

Tedersoo ord. nov.

180EF327-4C34-574C-B09B-21F423A4C97D

853580

##### Type family.

Kungsaengenaceae Tedersoo.

##### Description.

Covers the monophyletic group in Endogonomycetes (Fig. 2). Phylogenetically delimited as the least inclusive clade covering sequence accessions EUK1603402 and EUK1602136 (Suppl. material 3).

##### Notes.

Recognised based on eDNA sequences only. Currently includes Kungsaengenaceae.

#### ﻿ Kungsaengenaceae

Taxon classificationFungiKungsaengenales

Tedersoo fam. nov.

87BBC899-4CD0-53E2-8551-701053D6C46F

853581

##### Type genus.

*Kungsaengena* Tedersoo.

##### Description.

Covers the monophyletic group in Kungsaengenales (Fig. 2). Phylogenetically delimited as the least inclusive clade covering sequence accessions EUK1603402 and EUK1602136 (Suppl. material 3).

##### Notes.

Recognised based on eDNA sequences only. Currently includes *Kungsaengena* and a genus-level unassigned species represented by sequence EUK0013897 (GSMc plot G2907, subtropical forest soil in Cuc Phuong, Viet Nam, 20.34902°N, 105.59649°E).

#### ﻿ Kungsaengena

Taxon classificationFungiKungsaengenalesKungsaengenaceae

Tedersoo gen. nov.

A71ACC98-9F38-590A-B2E6-17C3DCF2E964

853582

##### Type species.

*Kungsaengenashadiae* Tedersoo.

##### Description.

Covers the monophyletic group in Kungsaengenaceae (Fig. 2). Phylogenetically delimited as the least inclusive clade covering sequence accessions EUK1603402 and EUK1602136 (Suppl. material 3).

##### Notes.

Based on ITS and LSU sequences, *Kungsaengena* comprises 5–6 species. Other putative species in this genus are represented by sequences EUK1603803 (GSMc plot G5906, stadium soil in Karksi-Nuia, Estonia, 58.10088°N, 25.55959°E); EUK1603124 (GSMc plot G5003, *Pinussylvestris* forest soil in Naissaar, Estonia, 59.5634°N, 24.5451°E); EUK1217319 (FunAqua sample W0279s, lake sediment near Bezdan, Serbia, 45.82031°N, 18.9599°E); and MW215857 (forest nursery soil in Lithuania).

#### ﻿ Kungsaengena shadiae

Taxon classificationFungiKungsaengenalesKungsaengenaceae

Tedersoo sp. nov.

4E91B05A-4FD2-5067-B51F-B76F2A2DE7F7

853583

##### Diagnosis.

**Figure 7.**
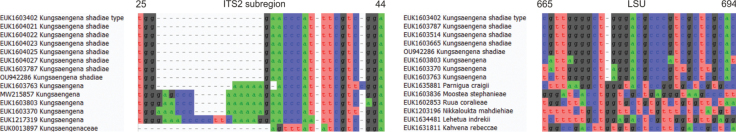
Diagnostic barcodes for *Kungsaengenashadiae* relative to closely-related taxa in ITS2 and LSU.

separation from other species of *Kungsaengena* based on the ITS region (ITS2 positions 25–44 tgggaacccatttcgtcgga; one mismatch allowed) and LSU (positions 665–694 cgttggggctgggacgcccgtcgctcgcac; one mismatch allowed) as indicated in Fig. 7.

##### Type.

eDNA sample TUE128324 (***holotype***); eDNA sequence EUK1603402 (***lectotype***); GSMc plot G5763, wet grassland (soil sample TUE028324) in Haage, Estonia, 58.35555°N, 26.61277°E).

##### Description.

other sequences: EUK1604022 (GSMc plot G5906, football field soil in Karksi-Nuia, Estonia, 58.10088°N, 25.55959°E); EUK1604023 (GSMc plot G5844, wet pasture soil in Tuhala, Estonia, 59.23003°N, 25.00283°E); EUK1604025 (GSMc plot G4444, Estonia, mixed forest soil in Altnurga, Estonia, 58.53676°N, 26.28321°E); and OU942286 (grassland soil in Kungsängen, Sweden, 59.837°N, 17.661°E), isolated by Shadi Eshghi Sahraei (Eshghi Sahraei et al. 2022).

##### Etymology.

*Kungsängen* (Swedish) refers to type locality; and *Shadi* (Persian) refers to the first name of Shadi Eshghi Sahraei who analysed materials collected from the type locality.

##### Notes.

Found from the Baltic States and Sweden, with ITS and LSU sequences differing up to 15% and 1%, respectively. The ITS region is infested with microsatellite-like regions and homopolymers, and many sequence variants have long deletions in multiple positions. *K.shadiae* seems to be generalist in terms of habitat type.

#### ﻿ Langduoales

Taxon classificationFungi

Tedersoo ord. nov.

D17889EB-3E73-5C99-A9AB-5605ED77DBE2

853584

##### Type family.

Langduoaceae Tedersoo.

##### Description.

Covers the monophyletic group in Endogonomycetes (Fig. 2). Phylogenetically delimited as the least inclusive clade covering sequence accessions EUK1107335, EUK1103607 and EUK1632831 (Suppl. material 3).

##### Notes.

Recognised based on eDNA sequences only. Currently includes Langduoaceae and another potentially family-level group, which is represented by sequences EUK1632831 (GSMc plot G4104, *Salixalba* wetland forest soil in Koiva, Estonia, 57.68283°N, 26.20146°E); EUK1603795 (GSMc plot G5906, football field in Karksi-Nuia, Estonia, 58.10088°N, 25.55959°E); and EUK1602996 (GSMc plot G4171, mixed coniferous forest soil in Nõmmeotsa, Estonia, 58.48765°N, 26.22523°E).

#### ﻿ Langduoaceae

Taxon classificationFungiLangduoales

Tedersoo fam. nov.

DC725A98-B736-55A5-8E83-D039AA3741FE

853585

##### Type genus.

*Langduoa* Tedersoo.

##### Description.

Covers the monophyletic group in Langduoales (Fig. 2). Phylogenetically delimited as the least inclusive clade covering sequence accessions EUK1107335, EUK1103607 and EUK1632829 (Suppl. material 3).

##### Notes.

Recognised based on eDNA sequences only. Currently represented by *Langduoa*.

#### ﻿ Langduoa

Taxon classificationFungiLangduoalesLangduoaceae

Tedersoo gen. nov.

E64A7959-C72F-5134-BD05-FAC855682359

853586

##### Type species.

*Langduoadianae* Tedersoo.

##### Description.

Covers the monophyletic group in Langduoaceae (Fig. 2). Phylogenetically delimited as the least inclusive clade covering sequence accessions EUK1107335, EUK1103607 and EUK1632829 (Suppl. material 3).

##### Notes.

Based on ITS sequences, *Langduoa* is comprised of 40–50 species. The genus is distributed globally in multiple habitat types, but not found from roots so far. Most *Langduoa* species are poorly separable based on the LSU marker. Other putative species in *Langduoa* are represented by sequences EUK1103607 (tropical rainforest soil in El Yunque, Puerto Rico, 18.29°N, -65.78°E); EUK1631446 (GSMc plot G4189, *Populustremula* forest soil in Tammsaare, Estonia, 57.84444°N, 27.20141°E); and MW215048 (tree nursery soil in Lithuania), which was recorded by Diana Marčiulynienė (Marčiulynienė et al. 2021).

#### ﻿ Langduoa dianae

Taxon classificationFungiLangduoalesLangduoaceae

Tedersoo sp. nov.

540EE31A-230A-5EA2-B48A-7CBFA29997CF

853587

##### Diagnosis.

**Figure 8.**
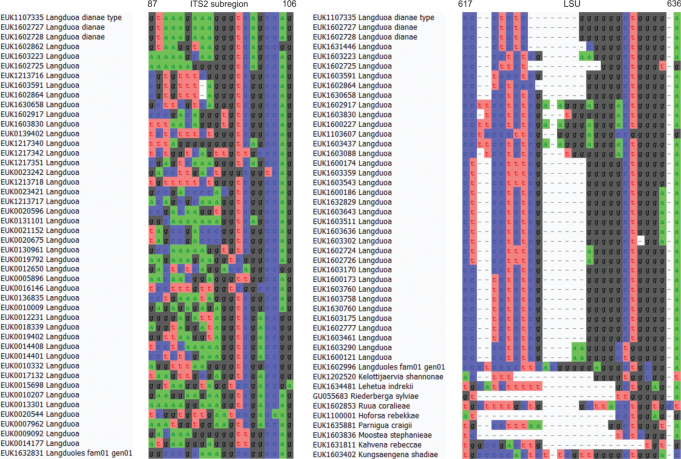
Diagnostic barcodes for *Langduoadianae* relative to closely-related taxa in ITS2 and LSU.

Separation from other species of *Langduoa* based on the ITS region (positions 87–106 actgagccttgcagcaacaatctccccttt; no mismatch allowed) and LSU (positions 617–636 ccctctcggggggctgggga; no mismatch allowed) as indicated in Fig. 8.

##### Type.

Soil eDNA sample TUE128827 (***holotype***); eDNA sequence: EUK1107335 (***lectotype***); montane grassland in Langduo, Tibet, 29.4°N, 94.4°E.

##### Description.

Other sequences: EUK1602727 and EUK1602728 (both from GSMc plot G5906, stadium grassland soil in Karksi-Nuia, Estonia, 58.10088°N, 25.55959°E); EUK1604031 (GSMc plot G4185, *Picea-Pinus* forest soil in Ristipalo, Estonia, 58.10241°N, 27.47874°E); and EUK1604032 (GSMc plot G4766, soil of coppiced garden dominated by *Fraxinus* and *Ulmus* in Ruudiküla, Estonia, 58.33630°N, 25.78084°E).

##### Etymology.

*Langduo* (Tibetan) refers to type locality; and *Diana* (Lithuanian) refers to the first name of Diana Marčiulynienė who was the first to record this genus.

##### Notes.

Found from grassland soils in Estonia and Tibet, with ITS and LSU sequences differing up to 0.2%. So far, not found from the roots.

#### ﻿ Lehetuales

Taxon classificationFungi

Tedersoo ord. nov.

E66B1F7F-6F8C-5AEC-9358-ADF9EF53D43F

853588

##### Type family.

Lehetuaceae Tedersoo.

##### Description.

Covers the monophyletic group in Endogonomycetes (Fig. 2). Phylogenetically delimited as the least inclusive clade covering sequence accessions EUK1603180, EUK1602375 and EUK1602377 (Suppl. material 3).

##### Notes.

Recognised based on eDNA sequences only. Currently includes Lehetuaceae.

#### ﻿ Lehetuaceae

Taxon classificationFungiLehetuales

Tedersoo fam. nov.

CAD10076-055E-5EC8-B4C1-E73F5316175F

853589

##### Type genus.

*Lehetua* Tedersoo.

##### Description.

Covers the monophyletic group in Lehetuales (Fig. 2). Phylogenetically delimited as the least inclusive clade covering sequence accessions EUK1603180, EUK1602375 and EUK1602377 (Suppl. material 3).

##### Notes.

Recognised based on eDNA sequences only. Currently includes *Lehetua* and another potentially genus-level group that is represented by sequences EUK1602869 (GSMc plot G4531, *Piceaabies* forest soil in Selisoo, Estonia, 57.621658°N, 27.179296°E) and EUK1603296 (GSMc plot S590, *Populustremula* forest soil in Lehetu, Estonia, 59.01857°N, 24.28041°E); and unassigned sequences EUK0025664 (GSMc plot G5536, tropical rainforest soil in Bamboesi, Suriname, 5.54086°N, -54.03131°E) and EUK0030289 (GSMc plot AV120, tropical rainforest soil in El Zafire, Colombia, -3.9997°N, 69.8947°E).

#### ﻿ Lehetua

Taxon classificationFungiLehetualesLehetuaceae

Tedersoo gen. nov.

6B88749C-F6F5-588C-A1D9-7124A2204C64

853590

##### Type species.

*Lehetuaindrekii* Tedersoo.

##### Description.

Covers the monophyletic group in Lehetuaceae (Fig. 2). Phylogenetically delimited as the least inclusive clade covering sequence accessions EUK1603180, EUK1602366 and EUK1602374 (Suppl. material 3).

##### Notes.

Based on ITS and LSU sequences, *Lehetua* is comprised of 8–10 species. Other putative ITS-based species in *Lehetua* are represented by sequences EUK1602811 (GSMc plot G4105, *Piceaabies* forest soil in Lepa, Estonia, 57.70158°N, 26.23993°E); EUK1603124 (GSMc plot G5003, *Pinussylvestris* forest soil in Naissaar, Estonia; 59.5634°N, 24.5451°E); and EUK0022184 (GSMc plot AV106, *Pseudomonotestropenbosii* rainforest soil in El Zafire, Colombia, -3.995°N, -69.898°E).

#### ﻿ Lehetua indrekii

Taxon classificationFungiLehetualesLehetuaceae

Tedersoo sp. nov.

EB330B0E-F11F-5A3C-AA19-7758D63BE4E1

853591

##### Diagnosis.

**Figure 9.**
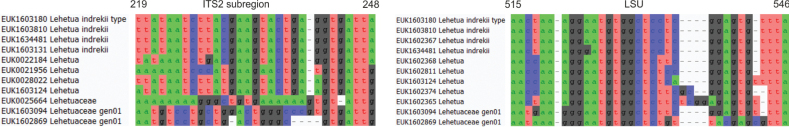
Diagnostic barcodes for *Lehetuaindrekii* relative to closely-related taxa in ITS2 and LSU.

Separation from other species of *Lehetua* based on the ITS region (positions 219–248 ttataatcttacgaagtactgaggtgatta; one mismatch allowed) and LSU (positions 515–546 aactaaaggratgtggctcctcggagtgttta; one mismatch allowed) as indicated in Fig. 9.

##### Type.

Soil eDNA sample TUE103095 (***holotype***); type sequence EUK1603180 (***lectotype***); GSMc plot S590, *Populustremula* forest (soil sample TUE003095) in Lehetu, Estonia, 59.01857°N, 24.28041°E.

##### Description.

Other sequences: EUK1603180 (type locality); EUK1602367 (LSU only; type locality; also found in 50 other sites in Estonia); EUK1634481 (GSMc plot G4195, *Quercusrobur* woodland soil in Lustivere, Estonia, 58.66293°N, 26.08465°E); EUK1603818 (GSMc plot G5824, managed grassland soil in Kuremaa, Estonia, 58.74138°N, 26.52727°E); EUK1603131 (GSMc plot G4105, *Piceaabies* forest soil in Lepa, Estonia, 57.70158°N, 26.23993°E); EUK0021956 (GSMc plot G5150, subarctic grassland soil in Kokelv, Norway, 70.61116°N, 24.62483°E); and EUK0023592 (GSMc plot S035, mixed deciduous forest soil in Kedrovaya Pad, Russia, 43.10834°N, 131.55447°E).

##### Etymology.

*Lehetu* (Estonian) refers to type locality (also meaning “leafless”); and *Indrek* (Estonian) refers to the first name of Indrek Hiiesalu who collected materials from the type locality.

##### Notes.

Found in Baltic States, Scandinavia and Russia, with ITS and LSU sequences differing up to 3.5% and 0.2%, respectively. Seems to be a generalist in terms of habitat type and soil pH; so far, not found from roots.

#### ﻿ Lokrumales

Taxon classificationFungi

Tedersoo ord. nov.

21ACC375-A4D8-5CC7-BA4A-8CED06A36780

853594

##### Type family.

Lokrumaceae Tedersoo.

##### Description.

Covers the monophyletic group in Endogonomycetes (Fig. 2). Phylogenetically delimited as the least inclusive clade covering sequence accessions EUK1203766, EUK1600125 and EUK1600268 (Suppl. material 3).

##### Notes.

Recognised based on eDNA sequences only. Currently includes Lokrumaceae and another potentially family-level taxon, represented by sequences EUK1602809 (GSMc plot G4499, rich, calcareous *Piceaabies* forest soil in Kurisoo, Estonia; 59.12808°N, 25.76395°E); EUK1603041 and EUK1603145 (both GSMc plot G4185, *Picea-Pinus* forest soil in Ristipalo, Estonia, 58.10241°N, 27.47874°E).

#### ﻿ Lokrumaceae

Taxon classificationFungiLokrumales

Tedersoo fam. nov.

EC7E1DA2-F922-56B7-BB63-8064778B6AB6

853595

##### Type genus.

*Lokruma* Tedersoo.

##### Description.

Covers the monophyletic group in Lokrumales (Fig. 2). Phylogenetically delimited as the least inclusive clade covering sequence accessions EUK1203766, EUK1600125 and EUK1600078 (Suppl. material 3).

##### Notes.

Recognised based on eDNA sequences only. Currently includes *Lokruma* and a few sequences not assigned to any genus; these include EUK0014543 and EUK0006923 (both GSMc plot G5106, subtropical forest soil in Brejo da Lapa, Brazil, -22.3582°N, -44.7383°E) and EUK1602939 (GSMc plot G4464, *Quercusrobur* forest soil in Ruu, Estonia, 59.45059°N, 25.22166°E).

#### ﻿ Lokruma

Taxon classificationFungiLokrumalesLokrumaceae

Tedersoo gen. nov.

118E7589-2DE2-5053-8FC7-469A0F7D1B6C

853596

##### Type species.

*Lokrumastenii* Tedersoo.

##### Description.

Covers the monophyletic group in Lokrumaceae (Fig. 2). Phylogenetically delimited as the least inclusive clade covering sequence accessions EUK1203766, EUK1600125 and EUK1600078 (Suppl. material 3).

##### Notes.

Based on ITS sequences, *Lokruma* is comprised of 35–40 species, some of which are represented by sequences EUK1200048 (GSMc plot G5130, grassland soil in Angera, Italy, 45.77336°N, 8.59657°E); EUK1602967 (GSMc plot G4626, *Picea-Populus* forest soil in Kõrve, Estonia, 59.07754°N, 26.76144°E); and EUK1603058 (*Piceaabies* forest soil in Serga, Estonia, 57.76052°N, 27.47502°E). Given the relatively high intraspecific differences and low interspecific differences, the LSU region is not optimal for distinguishing species of *Lokruma*.

#### ﻿ Lokruma stenii

Taxon classificationFungiLokrumalesLokrumaceae

Tedersoo sp. nov.

76664418-0381-5937-993C-E8A1D5B82667

853597

##### Diagnosis.

**Figure 10.**
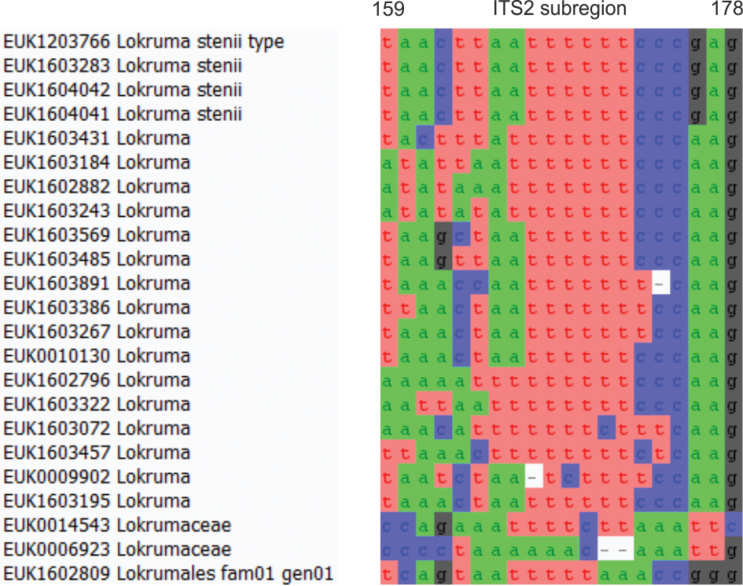
Diagnostic barcodes for *Lokrumastenii* relative to closely-related taxa in ITS2.

Separation from other species of *Lokruma* based on the ITS region (positions 159–178 taacttaattttttcccgag; one mismatch allowed) as shown in Fig. 10. There are no short barcodes in the first 700 bp of LSU that allow distinguishing *L.stenii* all from other congeners.

##### Type.

Soil eDNA sample TUE103193 (***holotype***); type sequence EUK1203766 (***lectotype***); GSMc plot S689, *Pinushalepensis* forest (soil sample TUE003193) in Lokrum, Croatia, 42.6223°N, 18.1241°E.

##### Description.

Other sequences: EUK1603283 (GSMc plot G4301, *Betulapendula* forest soil in Männamaa, Estonia, 58.83258°N, 22.63346°E); EUK1604041 (GSMc plot S480, *Populus-Picea* forest soil in Käru, Estonia, 58.80407°N, 25.22249°E); EUK1604042 (GSMc plot G4734, *Populus-Alnus* forest soil in Urissaare, Estonia, 58.02673°N, 24.65739°E); and EUK1600039 (LSU: GSMc plot HB19, *Populusxwettsteinii* forest plantation soil, Oja, Estonia, 58.82747°N, 26.37799°E).

##### Etymology.

*Lokrum* (Serbo-Croatian) refers to type locality; and *Sten* (Estonian) refers to the first name of Sten Anslan who collected the materials from the type locality.

##### Notes.

Found in Croatia and Estonia, with ITS and LSU sequences displaying up to 1% of differences.

#### ﻿ Moosteales

Taxon classificationFungi

Tedersoo ord. nov.

253AD908-CEEC-516A-9648-8AF7F4409EF3

853598

##### Type family.

Moosteaceae Tedersoo.

##### Description.

Covers the monophyletic group in Endogonomycetes (Fig. 2). Phylogenetically delimited as the least inclusive clade covering sequence accessions EUK1604044, JQ311412 and EUK1600278 (Suppl. material 3).

##### Notes.

Recognised based on eDNA sequences only. Currently includes Moosteaceae.

#### ﻿ Moosteaceae

Taxon classificationFungiMoosteales

Tedersoo fam. nov.

6C93DE73-BECB-5482-8FDE-AC38CD9FDEE3

853600

##### Type genus.

*Moostea* Tedersoo.

##### Description.

Covers the monophyletic group in Moosteales (Fig. 2). Phylogenetically delimited as the least inclusive clade covering sequence accessions EUK1604044, JQ311412 and EUK1600278 (Suppl. material 3).

##### Notes.

Recognised based on eDNA sequences only. Currently includes *Moostea* and two other potential genera. One of these is represented by sequences EUK0030179 (GSMc plot G4146, mixed forest soil in High Point Reserve Park, NJ, USA, 41.31569°N, -74.66485°E); EUK1600279 (GSMc plot G5826, *Malusdomestica* orchard soil in Tabivere, Estonia, 58.54286°N, 26.61575°E); and JQ311412 (microcosm soil in Los Alamos, NM, USA), isolated by Stephanie A. Eichorst (Eichorst and Kuske 2012). The other genus is represented by sequences EUK1600278 (GSMc plot S570, *Betulapubescens* wetland forest soil in Nõmme, Estonia, 58.47962°N, 22.94584°E); EUK0029679 (GSMc plot G2749, *Eucalyptus* spp. woodland soil near Lake Copperfield, Australia, -13.84191°N, 131.81858°E); and EUK0028885 (GSMc plot G5081, *Coccoloba* sp. woodland soil near Lagoa Grande, Brazil, -10.6342°N, -36.7579°E).

#### ﻿ Moostea

Taxon classificationFungiMoostealesMoosteaceae

Tedersoo gen. nov.

72EA30F4-A8C0-596C-A6E9-85F1EAE8E3B5

853601

##### Type species.

*Moosteastephanieae* Tedersoo.

##### Description.

Covers the monophyletic group in Moosteaceae (Fig. 2). Phylogenetically delimited as the least inclusive clade covering sequence accessions EUK1604044, EUK1103239 and EUK1600287 (Suppl. material 3).

##### Notes.

The ITS sequences are poorly alignable because of long deletions and inserts in certain species. Based on ITS sequences, *Moostea* is comprised of 25–30 species, some of which are represented by sequences EUK1103239 (tropical rainforest soil in El Yunque, Puerto Rico, 18.29°N, -65.78°E); EUK1603515 (GSMc plot G5835, airfield soil in Ridali, Estonia, 57.93692°N, 26.98099°E); and EUK0014332 (GSMc plot S1225, grassland soil in Ayapel, Colombia, 8.27825°N, -75.1257°E).

#### ﻿ Moostea stephanieae

Taxon classificationFungiMoostealesMoosteaceae

Tedersoo sp. nov.

0EF302DD-BD47-544E-A9A2-605C87529DC0

853603

##### Diagnosis.

**Figure 11.**
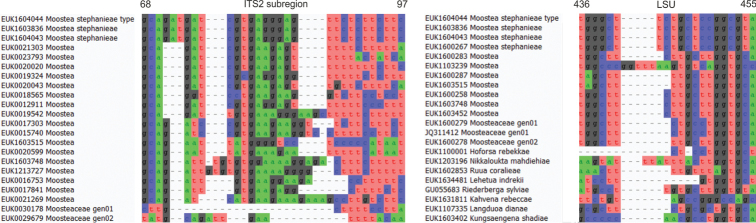
Diagnostic barcodes for *Moosteastephanieae* relative to closely-related taxa in ITS2 and LSU.

Separation from other species of *Moostea* based on the ITS region (positions 68–97 gcagatgatcgtgagggagttctcttcttc; one mismatch allowed) and LSU (positions 436–455 tgggcttctgctccggcgta; one mismatch allowed) as indicated in Fig. 11.

##### Type.

Soil eDNA sample TUE128417 (***holotype***); eDNA sequence EUK1604044 (***lectotype***); GSMc plot G5828, *Malusdomestica* orchard (soil sample TUE028417) in Mooste, Estonia, 58.15335°N, 27.19642°E.

##### Description.

Other sequences: EUK1600287 (LSU: type locality); EUK1604043 and EUK1603823 (both GSMc plot G5835, airfield soil in Ridali, Estonia, 57.93692°N, 26.98099°E).

##### Etymology.

*Mooste* (Estonian) refers to type locality; and *Stephanie* (English) refers to the first name of Stephanie A. Eichorst who collected the first materials from the respective family.

##### Notes.

Found in two sites in Estonia, with ITS and LSU sequences displaying up to 1% and 0.3% differences, respectively.

#### ﻿ Nikkaluoktales

Taxon classificationFungi

Tedersoo ord. nov.

57DD4DAD-2A4B-500C-8AB0-FBBC3019945B

853604

##### Type family.

Nikkaluoktaceae Tedersoo.

##### Description.

Covers the monophyletic group in Endogonomycetes (Fig. 2). Phylogenetically delimited as the least inclusive clade covering sequence accessions EUK1203196, EUK1600291 and EUK1600248 (Suppl. material 3).

##### Notes.

Recognised based on eDNA sequences only. Currently includes Nikkaluoktaceae.

#### ﻿ Nikkaluoktaceae

Taxon classificationFungiNikkaluoktales

Tedersoo fam. nov.

D9F0E42A-593A-5E2A-AD53-C7C9823073F6

853605

##### Type genus.

*Nikkaluokta* Tedersoo.

##### Description.

Covers the monophyletic group in Nikkaluoktales (Fig. 2). Phylogenetically delimited as the least inclusive clade covering sequence accessions EUK1203196, EUK1600291 and EUK1600248 (Suppl. material 3).

##### Notes.

Recognised based on eDNA sequences only. Currently includes *Nikkaluokta* and another potentially genus-level group that is represented by sequences EUK1602730 (GSMc plot S554, *Betula-Quercus* woodland soil in Mädapea, Estonia, 59.32169°N, 26.2621°E); EUK1602729 (GSMc plot FF14, *Piceaabies* forest soil in Kõdesi, Estonia, 58.61484°N, 27.12781°E); and EUK1600257 (GSMc plot G4464, *Quercusrobur* forest soil in Ruu, Estonia, 59.45059°N, 25.22166°E).

#### ﻿ Nikkaluokta

Taxon classificationFungiNikkaluoktalesNikkaluoktaceae

Tedersoo gen. nov.

5DE29B8F-AEEF-59BB-8DB1-3345828F9AE4

853606

##### Type species.

*Nikkaluoktamahdiehiae* Tedersoo.

##### Description.

Covers the monophyletic group in Nikkaluoktales (Fig. 2). Phylogenetically delimited as the least inclusive clade covering sequence accessions EUK1203196, EUK1600291, EUK1600289, EUK1600235, EUK1600225, EUK1600250 and EUK1600248 (Suppl. material 3).

##### Notes.

Based on ITS and LSU sequences, *Nikkaluokta* is comprised of 15–20 species, some of which are represented by sequences EUK1603884 (GSMc plot G4406, mixed coniferous forest soil in Tarumaa, Estonia, 59.20745°N, 27.15333°E); EUK1603411 (GSMc plot G4462, *Salixviminalis* energy plantation soil in Kambja, Estonia, 58.25166°N, 26.71276°E); and EUK0006485 (GSMc plot MX23, *Pinushartwegii* montane forest soil in Iztaccihuatl, Mexico, 19.12622°N, -98.65972°E).

#### ﻿ Nikkaluokta mahdiehiae

Taxon classificationFungiNikkaluoktalesNikkaluoktaceae

Tedersoo sp. nov.

A2004B52-C41B-510F-93D8-14C7C8D1AD94

853607

##### Diagnosis.

**Figure 12.**
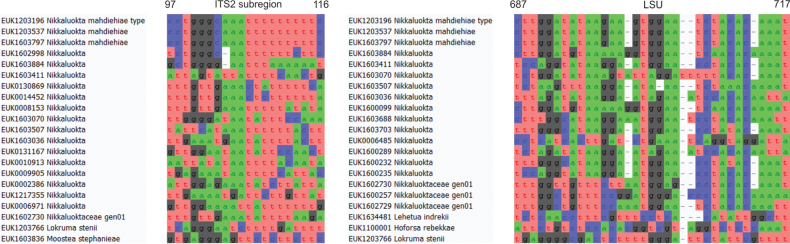
Diagnostic barcodes for *Nikkaluoktamahdiehiae* relative to closely-related taxa in ITS2 and LSU.

Separation from other species of *Nikkaluokta* based on the ITS region (positions 97–116 cctgggcaaatttttttttc; one mismatch allowed) and LSU (positions 687–717 cttggatataagaagtggaatctacacaaat; one mismatch allowed) as indicated in Fig. 12.

##### Type.

Soil eDNA sample TUE100497 (***holotype***); eDNA sequence EUK1203196 (***lectotype***); subarctic *Pinussylvestris* forest (soil sample TUE000497) in Nikkaluokta, Sweden, 67.85596°N, 19.47575°E.

##### Description.

Other sequences: EUK1203537 (type locality) and EUK1603797 (GSMc plot G5003, *Pinussylvestris* forest soil in Naissaare, Estonia, 59.56340°N, 24.54510°E).

##### Etymology.

*Nikkaluokta* (Sami) refers to type locality; and *Mahdieh* (Persian) refers to the first name of Mahdieh Hosseyni Moghaddam who sequenced the type materials using target capture protocols.

##### Notes.

Found in Sweden and Estonia, with ITS and LSU sequences displaying up to 1% and 0.2% differences, respectively.

#### ﻿ Parniguales

Taxon classificationFungi

Tedersoo ord. nov.

84530192-F9FD-5536-B614-A92CE860FCCC

853608

##### Type family.

Parniguaceae Tedersoo.

##### Description.

Covers the monophyletic group in Endogonomycetes (Fig. 2). Phylogenetically delimited as the least inclusive clade covering sequence accessions EUK1635261, EUK1602353, EUK1602857 and EUK1602732 (Suppl. material 3).

##### Notes.

Recognised based on eDNA sequences only. Currently represented by Parniguaceae.

#### ﻿ Parniguaceae

Taxon classificationFungiParniguales

Tedersoo fam. nov.

6227D941-6F9F-5139-8DEE-A97F9BE5A65B

853609

##### Type genus.

*Parnigua* Tedersoo.

##### Description.

Covers the monophyletic group in Parniguales (Fig. 2). Phylogenetically delimited as the least inclusive clade covering sequence accessions EUK1635261, EUK1602353, EUK1602857 and EUK1602732 (Suppl. material 3).

##### Notes.

Recognised based on eDNA sequences only. Currently represented by *Parnigua* and another potentially genus-level group, which is characterised by sequences EUK0016514 (GSMc plot S1218, urban park soil in Qujing, China, 25.52619°N, 103.74497°E), EUK0028452 (GSMc plot G3060, *Vateriaindica* forest in Hebri, India, 13.45437°N, 75.02213°E), EUK1602857 (GSMc plot G5771, grassland soil in Hino, Estonia, 57.57566°N, 27.22649°E), EUK1602732 (GSMc plot G5777, grassland soil in Eoste, Estonia, 58.11427°N, 27.08404°E) and EUK1602733 (GSMc plot G5816, *Trifoliumpratense* cropland soil in Hermani, Estonia, 58.80705°N, 25.75639°E).

#### ﻿ Parnigua

Taxon classificationFungiParnigualesParniguaceae

Tedersoo gen. nov.

DCC9362C-CDEF-56DD-BFCC-23F4F2B5B9C2

853610

##### Type species.

*Parniguacraigii* Tedersoo.

##### Description.

Covers the monophyletic group in Parniguaceae (Fig. 2). Phylogenetically delimited as the least inclusive clade covering sequence accessions EUK1635261 and EUK1602353 (Suppl. material 3).

##### Notes.

Based on stringent criteria, there are around five species in this genus, but all these may represent a single variable biological species. In this genus, across and within species, the ITS region has very low variability when compared with LSU (up to 3% differences across species). Other putative species in *Parnigua* are represented by sequences EUK1602947 (GSMc plot G4444, mixed forest soil in Altnurga, Estonia, 58.53676°N, 26.28321°E); EUK1603686 (GSMc plot G5844, wet pasture land soil in Tuhala, Estonia, 59.23003°N, 25.00283°E); EUK1633696 (GSMc plot G4207 *Tiliacordata* forest soil in Ubari, Estonia, 59.492609°N, 25.285663°E); EUK1603848 (GSMc plot G5883, flooded grassland soil in Kasari, Estonia, 58.73608°N, 23.98599°E); EUK1602353 (GSMc plot G4389, *Quercus-Tilia* forest soil in Naha, Estonia, 57.520914°N, 26.601199°E); MF484762 (agricultural soil in England); and MW163928 (*Crocussativus* cropland soil in Aosta Valley, Italy). The genus can be found from various soils but not from roots. However, SSU sequences are lacking, and links to AM fungi in SSU-based studies cannot be tested.

#### ﻿ Parnigua craigii

Taxon classificationFungiParnigualesParniguaceae

Tedersoo sp. nov.

EB911EB9-126A-5830-815B-5352D47ACEA6

853611

##### Diagnosis.

**Figure 13.**
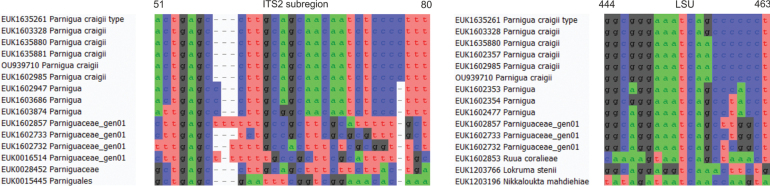
Diagnostic barcodes for *Parniguacraigii* relative to closely-related taxa in ITS2 and LSU.

Separation from other species of *Parnigua* based on the ITS region (positions 51–80 actgagccttgcagcaacaatctccccttt; no mismatch allowed) and LSU (positions 444–463 ggcgggaaatcagcccccct; no mismatch allowed) as indicated in Fig. 13.

##### Type.

Soil eDNA sample TUE102228 (***holotype***); type sequence: EUK1635261 (***lectotype***); GSMc plot G5251, *Quercusrobur* woodland (soil sample TUE002228) in Parnigu, Estonia, 58.64096°N, 26.38468°E.

##### Description.

Other sequences: EUK1635874 (GSMc plot G4499, calcareous *Piceaabies* forest soil in Kurisoo, Estonia; 59.12808°N, 25.76395°E); EUK1635875 (GSMc plot G4746, *Betulapendula* forest soil in Karjamõisa, Estonia, 57.59761°N, 26.35493°E); EUK1635878 (GSMc plot G4794, *Ulmus-Fraxinus* forest soil in Lõhtsuu, Estonia, 57.91781°N, 26.52069°E); EUK1603328 (GSMc plot G4167, *Salixpentandra* peat soil in Tammispää, Estonia, 58.92051°N, 27.01118°E); EUK1602985 (GSMc plot G5923, *Malusdomestica* orchard soil in Kalnabeites, Latvia, 57.1333°N, 24.8566°E); OU939710 (grassland soil in Kungsängen, Sweden, 59.837°N, 17.661°E); and MH625006 (grassland soil in Wakanui, New Zealand, -43.668°N, 172.470°E), first isolated by Craig R. Anderson (Anderson et al. 2018).

##### Etymology.

*Parnigu* (Estonian) refers to type locality; and *Craig* (English) refers to the first name of Craig R. Anderson who was the first to record this species.

##### Notes.

Found from Estonia, Sweden and New Zealand, with ITS and LSU sequences differing up to 0.5%. Found in all croplands, grasslands, deciduous and coniferous forests.

#### ﻿ Riederbergales

Taxon classificationFungi

Tedersoo ord. nov.

4E815FED-5175-5ABF-BDE7-D2E8BCEEDB04

853612

##### Type family.

Riederbergaceae Tedersoo.

##### Description.

Covers the monophyletic group in Endogonomycetes (Fig. 2). Phylogenetically delimited as the least inclusive clade covering sequence accessions EUK1602903, EUK1603115, EUK1602258, EUK1602253, EUK1602251 and EUK1104709 (Suppl. material 3).

##### Notes.

Recognised based on eDNA sequences only. Currently includes Riederbergaceae and four additional potentially family-level taxa represented by sequences EUK1100540 (bog peat soil in Svartberget, Sweden, 64.24°N, 19.76°E); EUK1602254 (GSMc plot G5826, *Malusdomestica* orchard in Tabivere, Estonia, 58.54286°N, 26.61575°E); EUK1602251, EUK1602253 and EUK1602257 (all GSMc plot G5828, Estonia, *Malusdomestica* orchard soil in Mooste, Estonia, 58.15335°N, 27.19642°E). Sequences EUK0031975 (GSMc plot S1082, *Araucariaaraucana* forest, Nahuelbuta, Chile, -37.78985°N, -73.0038°E) and EUK1217433 (GSMc plot G4777, maritime grassland (saltmarsh) soil in Härs-hämani, Estonia, 59.33103°N, 23.92720°E) represent additional, monospecific, potentially family-level groups not included in the phylograms due to the lack of LSU sequences.

#### ﻿ Riederbergaceae

Taxon classificationFungiRiederbergales

Tedersoo fam. nov.

45351A61-6079-5AB7-84BC-A513AA72CD3E

853613

##### Type genus.

*Riederberga* Tedersoo.

##### Description.

Covers the monophyletic group in Riederbergales (Fig. 2). Phylogenetically delimited as the least inclusive clade covering sequence accessions EUK1602903, EUK1602242 and EUK1602243 (Suppl. material 3).

##### Notes.

Recognised based on eDNA sequences only. Currently includes *Riederberga*.

#### ﻿ Riederberga

Taxon classificationFungiRiederbergalesRiederbergaceae

Tedersoo gen. nov.

2EEC473B-405C-52F3-AFFE-F114FBBC1D31

853614

##### Type species.

*Riederbergasylviae* Tedersoo.

##### Description.

Covers the monophyletic group in Riederbergaceae (Fig. 2). Phylogenetically delimited as the least inclusive clade covering sequence accessions EUK1602903, EUK1602242 and EUK1602243 (Suppl. material 3).

##### Notes.

Based on ITS and LSU sequences, *Riederberga* is comprised of 5–6 species, some of which are represented by sequences EUK1602859 (GSMc plot G4770, *Populusberolinensis* dominated coppiced garden in Ubasalu, Estonia, 59.06755°N, 24.47842°E); EUK1602912 (GSMc plot G4772, *Juniperuscommunis* calcareous woodland soil in Kohatu, Estonia, 58.95934°N, 24.30017°E); EUK1602761 (GSMc plot G4434, mixed woodland soil in Kalli, Estonia, 58.53770°N, 24.06659°E); and EUK1603687 (GSMc plot G4229, *Quercusrobur* woodland soil in Niidiaia, Estonia, 58.88603°N, 24.47280°E).

#### ﻿ Riederberga sylviae

Taxon classificationFungiRiederbergalesRiederbergaceae

Tedersoo sp. nov.

9E7F08B8-4B5F-5D4A-A6FD-E53BEF71D3D8

853615

##### Diagnosis.

**Figure 14.**
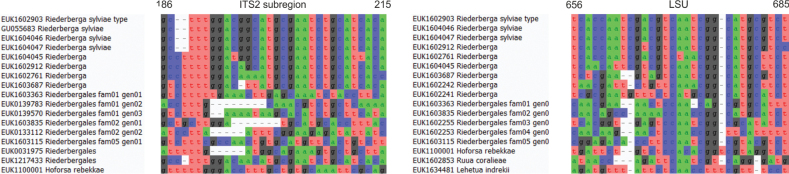
Diagnostic barcodes for *Riederbergasylviae* relative to closely-related taxa in ITS2 and LSU.

Separation from other species of *Riederberga* based on the ITS region (ITS2 positions 186–215 gctttggacggcatgcgaatctgcatcaca; one mismatch allowed) and LSU (positions 656–685 tcaccaatcgacgtcaatcggcatgcgtct; one mismatch allowed) as indicated in Fig. 14.

##### Type.

Soil eDNA sample TUE128372 (***holotype***); eDNA sequence: EUK1602903 (***lectotype***); GSMc plot G5783, wet grassland (soil sample TUE028372) in Altnurga, Estonia, 58.55682°N, 26.29259°E.

##### Description.

Other sequences: EUK1604046 and EUK1604047 (both type locality); and GU055683 (ITS part considered; managed grassland soil in Riederberg, Austria, 48.25°N, 16.07°E), collected by Sylvia Klaubauf (Klaubauf et al. 2010).

##### Etymology.

*Riederberg* (German) refers to type locality; and *Sylvia* (German) refers to the first name of Sylvia Klaubauf, who first collected the materials of type species and the entire order from the type habitat.

##### Notes.

Found in Austria and Estonia, with ITS and LSU sequences displaying up to 1% differences.

#### ﻿ Ruuales

Taxon classificationFungi

Tedersoo ord. nov.

F93E6A4C-D77E-56AB-BB21-164B965DB770

853616

##### Type family.

Ruuaceae Tedersoo.

##### Description.

Covers the monophyletic group in Endogonomycetes (Fig. 2). Phylogenetically delimited as the least inclusive clade covering sequence accessions EUK1603424, EUK1600239, EUK1600169 and EUK1600180 (Suppl. material 3).

##### Notes.

Recognised based on eDNA sequences only. Currently includes Ruuaceae.

#### ﻿ Ruuaceae

Taxon classificationFungiRuuales

Tedersoo fam. nov.

A5F825F3-3F11-54D4-BC46-D16EE26D497C

853617

##### Type genus.

*Ruua* Tedersoo.

##### Description.

Covers the monophyletic group in Ruuales (Fig. 2). Phylogenetically delimited as the least inclusive clade covering sequence accessions EUK1603424, EUK1600239, EUK1600169 and EUK1600180 (Suppl. material 3).

##### Notes.

Recognised based on eDNA sequences only. Currently includes *Ruua* and another genus-level taxon represented by sequence EUK1602764 (GSMc plot G4189, *Populustremula* forest soil in Tammsaare, Estonia, 57.84444°N, 27.20141°E).

#### ﻿ Ruua

Taxon classificationFungiRuualesRuuaceae

Tedersoo gen. nov.

12C8B808-14E0-5604-BAF2-30B66BF99003

853618

##### Type species.

*Ruuacoralieae* Tedersoo.

##### Description.

Covers the monophyletic group in Ruuaceae (Fig. 2). Phylogenetically delimited as the least inclusive clade covering sequence accessions EUK1603424, EUK1600239, EUK1600169 and EUK1600180 (Suppl. material 3).

##### Notes.

Based on ITS and LSU sequences, *Ruua* is comprised of 3–4 potential species that are represented by sequences EUK1632165 (GSMc plot S510, village habitat soil in Kihnu, Estonia, 58.1282°N, 23.9815°E); EUK1603289 (GSMc plot G4450, *Fraxinus-Tilia* forest soil in Nigula, Estonia, 58.0190°N, 24.6803°E); EUK1103406 (freshwater in Skogaryd, Sweden, 58.37°N, 12.16°E); and FN610984 (*Fagussylvatica* forest soil in Breuil-Chenue, France, 47.301°N, 4.076°E), isolated by Coralie Damon (Damon et al. 2010).

#### ﻿ Ruua coralieae

Taxon classificationFungiRuualesRuuaceae

Tedersoo sp. nov.

857DC242-41B5-5BCD-9ACA-B042FC3E784D

853619

##### Diagnosis.

**Figure 15.**
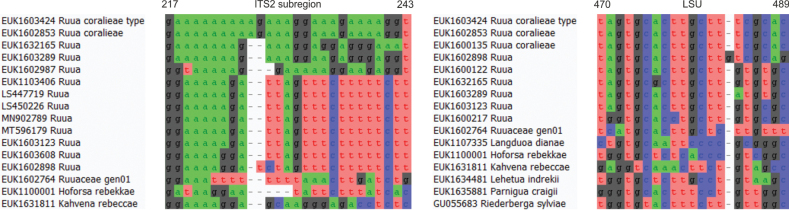
Diagnostic barcodes for *Ruuacoralieae* relative to closely-related taxa in ITS2 and LSU.

Separation from other species of *Ruua* based on the ITS region (positions 217–243 gaaaaaaaaagaaaggaaagaaaaggt; one mismatch allowed) and LSU (positions 470–489 tagtgcacttgctttcgcac; no mismatch allowed) as indicated in Fig. 15.

##### Type.

eDNA sample TUE101598 (***holotype***); eDNA sequence EUK1603424; GSMc plot G4464, *Quercusrobur* forest (soil sample TUE101598) in Ruu, Estonia, 59.45059°N, 25.22166°E.

##### Description.

Other sequences: EUK1602853 and EUK1600135 (type locality); EUK1604050 (GSMc plot G5002, *Tilia-Quercus* forest soil in Naissaar, Estonia, 59.57530°N, 24.53590°E); and EUK1604051 (GSMc plot S480, *Populus-Picea* forest soil in Käru, Estonia, 58.80407°N, 25.22249°E).

##### Etymology.

*Ruu* (Estonian) refers to type locality; and *Coralie* (French) refers to the first name of Coralie Damon, who collected the first materials belonging to this genus.

##### Notes.

Found from three sites in Estonia, with ITS and LSU sequences displaying up to 0.3% differences.

#### ﻿ Tammsaareales

Taxon classificationFungi

Tedersoo ord. nov.

95DA88D2-FCCD-598F-A206-6AC87C816BAB

853620

##### Type family.

Tammsaareaceae Tedersoo.

##### Description.

Covers the monophyletic group in Endogonomycetes (Fig. 2). Phylogenetically delimited as the least inclusive clade covering sequence accessions EUK1602762, EUK1635767 and EUK1602763 (Suppl. material 3).

##### Notes.

Recognised based on eDNA sequences only. Currently includes Tammsaareaceae.

#### ﻿ Tammsaareaceae

Taxon classificationFungiTammsaareales

Tedersoo fam. nov.

F9F1308F-B1A7-54BA-9336-11364D537E08

853621

##### Type genus.

*Tammsaarea* Tedersoo.

##### Description.

Covers the monophyletic group in Tammsaareales (Fig. 2). Phylogenetically delimited as the least inclusive clade covering sequence accessions EUK1602762, EUK1635767 and EUK1602763 (Suppl. material 3).

##### Notes.

Recognised based on eDNA sequences only. Currently includes *Tammsaarea* and the sequence EUK1602763 (GSMc plot G5835, airfield soil in Ridali, Estonia, 57.93692°N, 26.98099°E).

#### ﻿ Tammsaarea

Taxon classificationFungiTammsaarealesTammsaareaceae

Tedersoo gen. nov.

04D6E4EC-698B-5CA4-9FE1-217FEDFA3169

853622

##### Type species.

*Tammsaareavivikae* Tedersoo.

##### Description.

Covers the monophyletic group in Tammsaareaceae (Fig. 2). Phylogenetically delimited as the least inclusive clade covering sequence accessions EUK1602762 and EUK1635767 (Suppl. material 3).

##### Notes.

Based on ITS sequences, *Tammsaarea* is comprised of two species; the other being represented by LSU sequences EUK1601269, EUK1635767 and EUK1635768 (all GSMc plot G4185, *Picea-Pinus* forest soil in Ristipalo, Estonia, 58.10241°N, 27.47874°E).

#### ﻿ Tammsaarea vivikae

Taxon classificationFungiTammsaarealesTammsaareaceae

Tedersoo sp. nov.

5A06EDBE-08CB-5D3E-BC1C-68891B139752

853683

##### Diagnosis.

**Figure 16.**
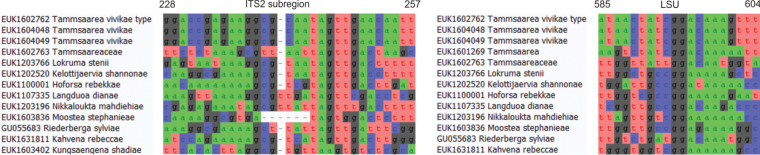
Diagnostic barcodes for *Tammsaareavivikae* relative to closely-related taxa in ITS2 and LSU.

Separation from other species of *Tammsaarea* and other species of Endogonomycetes based on ITS (positions 228–257 ggaccgagaaggcgcaatagttgaacaatt; one mismatch allowed) and LSU (positions 585–604 ataactatcggacaaagttt; one mismatch allowed) as indicated in Fig. 16.

##### Type.

eDNA sample TUE100731 (***holotype***); eDNA sequence EUK1602762 (***lectotype***); GSMc plot G4189, *Populustremula* forest (soil sample TUE000731) in Tammsaare, Estonia, 57.84444°N, 27.20141°E.

##### Description.

Other sequences EUK1604048 and EUK1604049 (type locality).

##### Etymology.

*Tammsaare* (Estonian) refers to the type locality and one of the most famous Estonian writers, Anton Hansen Tammsaare; and *Vivika* (Estonian) refers to the first name of Vivika Adamson who provided access to the type locality.

##### Notes.

Found from a single locality in Estonia, with ITS and LSU sequences differing up to 0.5% and 0.3%, respectively.

#### ﻿ Unemaeeales

Taxon classificationFungi

Tedersoo ord. nov.

826EEC8F-A6E8-55EB-AC2E-0EEBCAE0171C

853684

##### Type family.

Unemaeeaceae Tedersoo.

##### Description.

Covers the monophyletic group in Endogonomycetes (Fig. 2). Phylogenetically delimited as the least inclusive clade covering sequence accessions EUK1630871 and EUK1635889 (Suppl. material 3).

##### Notes.

Recognised based on eDNA sequences only. Currently includes Unemaeeaceae.

#### ﻿ Unemaeeaceae

Taxon classificationFungiUnemaeeales

Tedersoo fam. nov.

97D136DC-C849-579B-AA7C-D1763568E03E

853685

##### Type genus.

*Unemaeea* Tedersoo.

##### Description.

Covers the monophyletic group in Unemaeeales (Fig. 2). Phylogenetically delimited as the least inclusive clade covering sequence accessions EUK1630871 and EUK1635889 (Suppl. material 3).

##### Notes.

Recognised based on eDNA sequences only. Currently includes *Unemaeea* and multiple poorly alignable ITS sequences with no LSU, for example EUK1217297 (FunAqua sample W0006s, lake sediment in Petrolandia, Brazil, -8.9908°N, -38.2251°E) and FJ528738 (*Araucaria* spp. plantation soil, Gadgarra, Australia, -17.1641°N, 145.6469°E) that was isolated by Nathalie J.A. Curlevski (Curlevski et al. 2010). It seems that several Unemaeeaceae spp. have preferential habitat in anoxic soils and sediments.

#### ﻿ Unemaeea

Taxon classificationFungiUnemaeealesUnemaeeaceae

Tedersoo gen. nov.

66AC7F74-232A-5273-99D5-FF5E6CA159D3

853686

##### Type species.

*Unemaeeanathalieae* Tedersoo.

##### Description.

Covers the monophyletic group in Unemaeeales (Fig. 2). Phylogenetically delimited as the least inclusive clade covering sequence accessions EUK1630871 and EUK1635889 (Suppl. material 3).

##### Notes.

Based on ITS and LSU sequences, *Unemaeea* is comprised of three species; others represented by sequences EUK1217289 (freshwater lake sediment near Bezdan, Serbia, 45.82031°N, 18.9599°E) and KX196132 (deciduous forest soil in Champaign County, IL, USA).

#### ﻿ Unemaeea nathalieae

Taxon classificationFungiUnemaeealesUnemaeeaceae

Tedersoo sp. nov.

89C10250-C799-54E9-97E2-E2AAFE6E3EF9

853687

##### Diagnosis.

**Figure 17.**
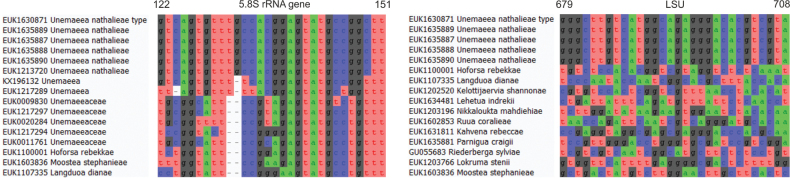
Diagnostic barcodes for *Unemaeeanathalieae* relative to closely-related taxa in ITS2 and LSU.

Separation from other species of *Unemaeea* based on the ITS region (5.8S positions 122–151 gtcagtgtttgccacggagtatgccggctt; no mismatch allowed) and from other species of Endogonomycetes based on LSU (positions 694–723 gggcttgtcatggcagagggacacgtcgta; no mismatch allowed) as indicated in Fig. 17.

##### Type.

Soil eDNA sample TUE100213 (***holotype***); eDNA sequence EUK1630871 (***lectotype***); GSMc plot G3318, marshland (soil sample TUE000213) in Unemäe, Estonia, 58.28253°N, 22.46296°E.

##### Description.

Other sequences: EUK1635887–EUK1635890 (type locality) and EUK1213720 (FunAqua sample W0581s, river sediment in Floresti, Romania, 46.75472°N, 23.49923°E).

##### Etymology.

*Unemäe* (Estonian) refers to the type locality; and *Nathalie* (English) refers to the first name of Nathalie J.A. Curlevski who collected the first materials belonging to this genus.

##### Notes.

The end of 5.8S and start of LSU are strongly diverged compared with other species of *Unemaeea* and Endogonomycetes. As no other confamilial LSU sequences are available, the diagnostic positions are compared against the most divergent, unalignable part across Endogonomycetes. Found in anoxic soil in Estonia and Romania, with ITS sequences displaying up to 4% differences.

#### ﻿ Bifiguratales

Taxon classificationFungi

Tedersoo ord. nov.

EEE1045F-7B8B-50D9-BA60-97274AFC4591

853688

##### Type family.

Bifigurataceae Tedersoo.

##### Description.

Covers the monophyletic group in Endogonomycetes (Fig. 2). Cultured mycelium filamentous, aseptate, coenocytic, 2 μm diam., mucose in appearance, commonly producing budding yeast-like cells; chlamydospores intercalary, 5–10 μm diam., forming on hyphal tips. Phylogenetically delimited by the least inclusive clade covering sequence accessions HM123225, EUK1104879, KF568171 and KF567389.

##### Notes.

Comprised of a single family Bifigurataceae. Order description is adapted from Torres-Cruz et al. (2017).

#### ﻿ Bifigurataceae

Taxon classificationFungiBifiguratales

Tedersoo fam. nov.

42100643-7102-5F0F-871D-8A0DB31BB616

853689

##### Type genus.

*Bifiguratus* T.J.Torres-Cruz & A.Porras-Alfaro.

##### Description.

Cultured mycelium filamentous, aseptate, coenocytic, 2 μm diam., mucose in appearance, commonly producing budding yeast-like cells; chlamydospores intercalary, 5–10 μm diam., forming on hyphal tips. Phylogenetically delimited by the least inclusive clade covering sequence accessions HM123225, EUK1104879, KF568171 and KF567389.

##### Notes.

Comprised of a single genus *Bifiguratus* that is commonly found in soil and occasionally in roots of non-AM plants. No sexual structures have been revealed. Family description is adapted from Torres-Cruz et al. (2017).

#### ﻿ Densosporales

Taxon classificationFungi

Tedersoo ord. nov.

861CE3AB-89AA-5874-BB2A-133E6C3004A1

853690

##### Type family.

Densosporaceae Desirò, M.E.Sm., Bidartondo, Trappe & Bonito.

##### Description.

Densosporales is defined as a monophyletic group in Endogonomycetes (Fig. 2, Suppl. material 3) that corresponds to Densosporaceae sensu Desiro et al. (2017). Covers *Densosporacae* and Planticonsortiaceae and the least inclusive clade with sequence accessions UDB028692, EUK1104889, EUK1104816 and EUK1601509 (Suppl. material 3).

##### Notes.

Densosporales harbours roughly one half of the Endogonomycetes based on LSU data. It comprises *Densosporacae*, Planticonsortiaceae and 16 additional family-level groups collectively covering >200 species. LSU has much greater phylogenetic resolution compared with SSU (Suppl. materials 3, 4), and the potential utility of the ITS region seems to vary greatly by family. Many more ITS-LSU sequences are needed to understand family- and genus-level composition of Densosporales.

#### ﻿ Densosporaceae

Taxon classificationFungiDensosporalesDensosporaceae

Desirò, M.E.Sm., Bidartondo, Trappe & Bonito, emend. Tedersoo

01C2B117-0490-54D9-9444-92E16980CE17

821851

##### Type genus.

*Densospora* McGee.

##### Description.

Phylogenetic diagnosis as in Desiro et al. (2017), but includes a more limited phylogenetic group - the least inclusive clade comprised of *Densospora* spp. (accessions JF414167 and UDB28692), *Sphaerocreaspubescens* (accession LC107618) and accession EUK1601029 (Suppl. material 3).

##### Notes.

Based on SSU phylogeny (Suppl. material 4), one or both of the genera *Densospora* and *Sphaerocreas* are paraphyletic, and their relationships require further research. Most species in both genera remain to be sequenced, including *D.tubiformis* (P.A.Tandy) McGee - the type species of *Densospora*.

#### ﻿ Planticonsortiaceae

Taxon classificationFungiDensosporales

Tedersoo fam. nov.

77431B6A-8B3B-5CCC-A716-2450B6002A98

853691

##### Type genus.

*Planticonsortium* C.Walker & D.Redecker.

##### Description.

Emanating hyphae 0.5–4 μm diam., forming colourless to brown chlamydospores (10–12 μm, up to 35 μm diam.), sometimes rope-like strands; appressoria swollen, frequently with several thin hyphae giving an insect-like appearance. Intraradical mycelium 0.5–4 μm diam., smooth to angular, with (sub-)globose swellings, forming comb-like (ctenoid), fan-shaped, palmate, antler-like, digitate or feather-like structures appearing clasped around epidermal and cortical cells; forming finely branched arbuscules. All hyphae stain darkly in acidic blue stains, more strongly for extraradical hyphae. Monophyletic group in Densosporales (Fig. 2, Suppl. materials 3, 4).

##### Notes.

Planticonsortiaceae covers roughly one third of Endogonomycetes reads based on LSU (Suppl. material 3) and SSU (Suppl. material 4), but is poorly represented in the ITS dataset. This may be due to the highly divergent and relatively long ITS region (800–1200 bases). Based on the LSU phylogram, Planticonsortiaceae harbours seven genus-level groups with >100 putative species. The description is adapted from Walker et al. (2018).

#### ﻿ Endogonales

Taxon classificationFungiEndogonalesEndogonaceae

Jacz. & P.A.Jacz., emend. Tedersoo

794A3239-8DBB-56F1-832D-0144835F524F

90720

##### Type family.

Endogonaceae Paol.

##### Description.

Fruiting body hypogeous or on debris, globose, irregular, sometimes resupinate, 1–10 mm in diam., may be composed of aggregated zygosporangial clusters, with zygospores formed on apposed suspensors. Hyphae of fruiting body tissue coenocytic, aseptate, sometimes with secondary septa that form micropores. Reproductive structures as zygosporangia, rarely azygosporangia (co-existing with zygosporangia in *Endogonepisiformis*) or chlamydospores (in *Vinositunica*), distributed randomly or radially in fruiting bodies, 100–700 μm diam., with yellow granular contents. Zygosporangial wall comprises outer sporangiothecium with 1–4 openings and inner eusporium with no openings. Azygosporangia rare, with a single-layered wall and separated from the single suspensor by a gametangial septum. Chlamydospore wall continuous, multilayered, with dense subtending hyphae, lacking septa. Forms a monophyletic group in Endogonomycetes as the least inclusive clade covering accessions EUK1601498, EUK1100757, LC002628, LC431107, EUK1104693 and UDB025468.

##### Notes.

Includes taxa with or without fruiting bodies and with ectomycorrhizal, arbuscular mycorrhizal and saprotrophic lifestyles. Endogonales harbours Endogonaceae, Jimgerdemanniaceae and Vinositunicaceae families, as well as seven potentially family-level taxa, collectively comprising >200 species based on ITS and LSU sequences. Order description is adapted from Morton and Benny (1990) and Yamamoto et al. (2020).

#### ﻿ Endogonaceae

Taxon classificationFungiEndogonalesEndogonaceae

Paol., emend. Tedersoo

B44E017E-4A61-5D53-B312-17EBAC90BB9D

81877

##### Type genus.

*Endogone* Link.

##### Description.

Fruiting body hypogeous or on debris, globose, irregular, sometimes resupinate, 1–10 mm diam., may be composed of aggregated zygosporangial clusters, with zygospores formed on apposed suspensors. Hyphae of fruiting body tissue coenocytic, aseptate, sometimes with secondary septa that form micropores. Reproductive structures as zygosporangia, rarely azygosporangia (co-existing with zygosporangia in *Endogonepisiformis*) distributed randomly or radially in fruiting bodies, 100–700 μm diam., with yellow granular contents. Zygosporangial wall comprises outer sporangiothecium with 1–4 openings and inner eusporium with no openings. Azygosporangia rare, with a single-layered wall, and separated from the single suspensor by a gametangial septum. Forms a monophyletic group in Endogonales as the least inclusive clade covering accessions LC002628, EUK1601764 and EUK1601442.

##### Notes.

Covers species of *Endogone* that are saprotrophic or potentially ectomycorrhizal (/endogone2 and /endogone3 lineages, *sensu*Tedersoo and Smith (2017)) and four closely-related genus-level taxa.

#### ﻿ Jimgerdemanniaceae

Taxon classificationFungiEndogonales

Tedersoo fam. nov.

9FA53440-1DC9-54F3-94C5-49B14EA58F10

853692

##### Type genus.

*Jimgerdemannia* Trappe, Desirò, M.E.Sm., Bonito & Bidartondo.

##### Description.

Includes *Jimgerdemannia* and closely-related genera that form a monophyletic group in Endogonales, with the least inclusive clade covering accessions KC568319, EUK1631035, JN890102, UDB025468 and OU942919 (Suppl. material 3).

##### Notes.

Jimgerdemanniaceae covers an ectomycorrhizal genus *Jimgerdemannia* and six genus-level taxa that are soil-inhabiting, potentially arbuscular mycorrhizal and probably not producing macroscopic fruiting bodies.

#### ﻿ Vinositunicaceae

Taxon classificationFungiEndogonales

Tedersoo fam. nov.

B3DEA742-7B7D-587B-839E-1117637F08A0

853693

##### Type genus.

*Vinositunica* Koh.Yamam., Degawa & A.Yamada.

##### Description.

Fruiting bodies epigeous or semi-hypogeous, reniform or irregular, often with a short stipe-like sterile base, 2–20 mm in diam. Peridium white, partly purple, in a single layer, composed of coenocytic aseptate hyphae. Gleba pale yellow to purplish-grey, composed of numerous radially or randomly distributed chlamydospores. Chlamydospores granular, with yellow contents, broadly ellipsoid, 50–700 μm diam, terminal on single subtending hypha. Cell wall composed of purplish to vinaceous outer layer and colourless inner layer.

##### Notes.

Vinositunicaceae includes the genus *Vinositunica*. This group has not been found from root or soil eDNA samples thus far, and ITS sequences are not available. Probably humus saprotrophs. Family description is adapted from Yamamoto et al. (2020).

### ﻿Primer bias

To evaluate whether some part of the dark diversity of putative AM fungi can be accounted for by primer bias as suggested for Glomeromycota (Kohout et al. 2014; van Geel et al. 2014; Seeliger et al. 2023), we tested the commonly used SSU, ITS and LSU primers for critical mismatches based on multiple sequence alignments. The AMV4.5NF (Sato et al. 2005) and AM-Sal-F (Seeliger et al. 2023) primers, proposed to cover both AM fungal groups, exhibited several (near-)terminal mismatches to many groups of Glomeromycota and one central and one near-terminal mismatch to many groups of Endogonomycetes. The FRE-F (Seeliger et al. 2023) primer had multiple mismatches to most target Endogonomycetes groups including a terminal mismatch to some groups. The reverse SSU primers AMDGR (Sato et al. 2005) and FRE-R (Seeliger et al. 2023) matched well with Glomeromycota, but had one or more (near)-terminal mismatches to several groups of Endogonomycetes. Regarding the ITS-LSU primers, ITS1F (Gardes and Bruns 1993), ITS1 (White et al. 1990), gITS7ngs and ITS4ngsUni (Tedersoo and Lindahl 2016) had single central mismatches to a few Glomeromycota and Endogonomycetes lineages, whereas ITS9munngs (Tedersoo and Lindahl 2016) had no mismatches. The fungi-specific primer ITS1catta (Tedersoo and Anslan 2019) had (near)-terminal mismatches to several minor lineages of both AM groups.

Of Glomeromycota-specific primers, wSSUmcf (Krüger et al. 2009) matched well to all target lineages. The primer wLSUmbr (Krüger et al. 2009) had one central mismatch to *Pervetustus* and Archaeosporales, suggesting a negligible bias.

Of Endogonomycetes-specific primers designed and tested initially, ITS3-End displayed mismatches to multiple groups, while LR3-End had 1–2 central mismatches to Jimgerdemanniaceae and terminal mismatches to Unemaeeaceae. For Endogonomycetes, we thus recommend use of universal forward primers gITS7ngs or LROR or the newly-designed LF350End (ccgatagcgaacaagtac; also amplifies many other fungi) in combination with the combination of reverse primers LR3-End2 (aycattahgycagcgacc; >99% of Endogonomycetes) and LR3-End2a (aycattahgycagccgtta; Unemaeeaceae). These primer pairs yield amplicons of 900–1200 bases, ca. 700 bases and ca. 400 bases, respectively. For simultaneous amplification of Glomeromycota and Endogonomycetes, only universal or fungal primers can be recommended (e.g., forward primers gITS7ngs, LROR and LF350 combined with a reverse primer TW13; White et al. (1990)) along with deep sequencing to 10^5^ reads.

## ﻿Discussion

In this paper, we describe 15 new species of potentially AM fungi belonging to Glomeromycota and Endogonomycetes from soil eDNA samples. These new species and six re-combinations lead to 16 new genera, 19 new families and 17 new orders that are well delimited by phylogenetic analyses of rRNA genes. The high taxonomic and phylogenetic resolution at the levels of species to class render long-read rRNA gene sequences highly useful for both species delimitation and phylogeny reconstruction. Future studies using protein-encoding genes or whole-genome analyses will be useful for solving phylogenetic uncertainties related to rapid rRNA gene evolution in certain groups (e.g. Entrophosporales) and unsettled branching order (e.g. endogonomycete orders). For this study, the genomes that were available for only 13 described genera of Glomeromycota and two genera of Endogonomycetes (Rosling et al. 2024) would have added no extra value. Our phylogenies indicate that eDNA from soil and sediment habitats may substantially add to novel phylogenetic diversity in these groups, especially in Endogonomycetes. Studies combining fine root staining and DNA sequencing should improve our understanding of the symbiotic potential of these newly-described groups and the evolution of AM associations in general.

We rely on public long-read rRNA gene sequences to describe new species in previously unrecognised family- and order-level taxa, using eDNA samples as holotypes and sequences as lectotypes. Previous DNA-based taxonomic studies on fungi have described new species in well-known genera (Bridge and Hughes 2012; Kirk 2012; Kalsoom Khan et al. 2020) or families (de Beer et al. 2016; Lücking and Moncada 2017) based on typifying sequences of the ITS region. The species described here are usually represented by both ITS and LSU regions from multiple eDNA samples. This allows us to estimate rough intraspecific variation and interspecific distances, and develop continuous, 20–30-base diagnostic barcodes (see also Kalsoom Khan et al. (2020)) for ITS and LSU regions separately. This contrasts with other studies that point to single diagnostic differences scattered across the entire marker length (de Beer et al. 2016; Lücking and Moncada 2017), or provide no sequence-diagnostic features. The continuous barcodes are better findable for the human eye and software, such as custom BLAST algorithms (Camacho et al. 2009), Cutadapt (Martin 2011), CAOS-R (Bergmann 2024) and SeqKit2 (Shen et al. 2024). For nearly all species (except *Parniguacraigii*), these diagnostic barcodes are more informative for the ITS region than LSU due to greater variability and taxonomic resolution. The species of *Langduoa* and *Lokruma* have relatively lower LSU short barcode resolution compared with other taxa. Nonetheless, species from all groups can be distinguished well based on ITS1 or ITS2 sequences and usually by LSU sequences.

The newly-described species, genera, families and orders are represented exclusively by eDNA sequences supplied with metadata ranging from none to ample background information about location and environmental properties, depending on the source of reads and success in contacting the data producers or material collectors. Besides fragmented information about habitat and distribution, soil eDNA provides no information about biotic interactions or functioning. Given the paucity of data from non-soil habitats outside northern Europe, we refrain from speculating about the distribution and functional role of the described species and higher-level taxa.

The Glomeromycota SSU-ITS-LSU phylogram is congruent with previous studies at the level of families and orders (Oehl et al. 2011; Redecker et al. 2013; Blaszkowski et al. 2021, 2022; Montoliu-Nerin et al. 2021; Rosling et al. 2024). The newly-described entrophosporalean family Pseudoentrophosporaceae does not affect the overall phylogenetic structure of Glomeromycota, but expands the phylogenetic breadth of Entrophosporales. Besides this new family, we also recorded multiple potentially new genera, most of which have also been revealed in previous analyses of root and soil materials. In this paper, we refrain from formally describing these for two main reasons. First, most of these genera are relatively common, and there are high chances that the corresponding species have been described but yet to be sequenced; here, we sincerely hope that the current study motivates the publishing of these materials, kept in several research teams’ drawers. Second, we are surveying hundreds of global soil and sediment samples using the ITS- and LSU-orientated Glomeromycota- and Endogonomycetes-specific primers, which will likely reveal novel diversity and improve delimiting the new putative taxa. For their short-term communication, we propose alphanumeric labels that facilitate quality-filtering, especially chimaera control, for forthcoming eDNA studies. The currently accepted genera and proposed genus-level groups for Glomeromycota and Endogonomycetes are provided in Suppl. materials 5, 6, respectively.

The Endogonomycetes SSU-5.8S-LSU phylogram only marginally reflects the SSU-focused multigene phylograms of Desiro et al. (2017) and Yamamoto et al. (2020) and the SSU-based phylogram of Albornoz et al. (2022). Here, we distinguish 17 well-supported orders in Endogonomycetes, including Densosporales and Bifiguratales (ord. nov.) and Endogonales (sens. str.), as well as entirely new groups represented by no sequenced culture, spore or fruiting body specimen. For anchoring names to these orders, we describe well-chosen representative species, genera and families based on eDNA and long-read sequences. To communicate these groups’ internal structure, we propose alphanumeric codes for putative families and genera as for the Glomeromycota. Furthermore, our analyses indicate much greater phylogenetic and species-level resolution for the LSU marker than the SSU (Suppl. materials 3, 4). The sequence data accumulated thus far also reveal much more information available for LSU compared with ITS and SSU at the level of species and orders (Desiro et al. 2017; Suppl. material 3). However, these datasets are biased for soil (ITS and LSU) or plant samples (SSU). As a downside of the ITS-LSU approach, the genus *Planticonsortium* lacks sequence data for these markers and cannot be reliably assigned to any of the multiple Planticonsortiaceae genus-level groups. However, ultra-long reads should be able to bridge the SSU and LSU and provide insights into Planticonsortiaceae soon.

We have encountered several conflicting situations by focusing on the mixed morphology- and eDNA-based classification. Undoubtedly, there is a potential risk of parallel morphological and DNA-based descriptions, especially given that nearly half of the accepted species of AM fungi are represented by no sequence data. However, the high-throughput sequencing methods have been available for >15 years, making it increasingly less likely that old spore collections from microscope slides will be successfully sequenced soon.

In addition to the parallel morphology-based and DNA-based descriptions, the focus on different morphological characters may also hamper the taxonomy of AM fungi. We find that a pair of glomeromycete genera, *Redeckera* and *Corymbiglomus*, that can be seemingly well delimited by morphological characters, are not clearly separated in phylogenetic analysis. Importantly, *Redeckera* spp. are described based on the small glomerospores clustering in large fruiting bodies, whereas species in *Corymbiglomus* are distinguished based on glomerospores on hyphal tips. The presence of spore dimorphism, as recently revealed for *Entrophospora*-*Claroideoglomus* (Blaszkowski et al. 2022), might be behind the inconsistency between phylogenetic and morphological data. Since Diversisporaceae harbours multiple described and undescribed genera, we leave the taxonomy of the *Redeckera-Corymbiglomus* group to be settled in further studies. Furthermore, species of Endogonomycetes have been described based on chlamydospores on hyphae (*Planticonsortium*), chlamydosporic fruiting bodies (*Vinositunica* and *Densospora*), zygosporangial fruiting bodies (*Endogone* and *Jimgerdemannia*) and features of pure cultures (*Bifiguratus*), potentially resulting in parallel classification based on different characters. Here, the massive amount of available eDNA sequences enables us to bridge a vast majority of these taxa (except *Vinositunica* and *Planticonsortium*) and translate the various types of morphological descriptions into a common DNA-based language.

## ﻿Conclusions

This study offers the first example of a mixed morphological and eDNA-based classification from species to order level in the fungal kingdom. Our approach of typifying both eDNA samples and sequences and preparing diagnoses based on DNA barcodes will likely boost alpha and higher-level taxonomic research in fungi and potentially in non-fungal organisms. Such a mixed classification would help provide human-readable names to many of the “dark matter” fungi (Nilsson et al. 2016) and tremendously reduce the number of entirely unidentified fungi. To avoid parallel DNA-based classifications, we propose that the description of new species should primarily focus on the universal fungal barcode - the ITS region, preferably supplemented with at least one additional taxonomically or phylogenetically informative marker - LSU or SSU in the case of amplicons - or a protein-encoding gene for genomes derived from tissues. Accordingly, we propose using both the ITS and LSU markers for the two groups of AM fungi, considering taxonomic resolution, availability of specific primers and the large number of previously described reference species.

Our research also points out that, in addition to registering newly-described fungal taxa, we urgently need a linked system (related to, for example, INSDC, MycoBank and/or UNITE - the leading platforms that cross-communicate fungal species and molecular sequence data) for mandatory registering of taxonomic emendations and taxonomic updates of sequences, especially when new taxa are erected based on already published sequences. Such sequence registration would minimise the risk that taxon names of the sequences in databases evolve in different directions and that new species are described several times based on the same or related sequences.

## Supplementary Material

XML Treatment for Diversispora bareae

XML Treatment for Diversispora nevadensis

XML Treatment for Diversispora

XML Treatment for Fuscutata cerradensis

XML Treatment for Fuscutata reticulata

XML Treatment for Viscospora deserticola

XML Treatment for Parvocarpum

XML Treatment for Parvocarpum badium

XML Treatment for Pseudoentrophosporaceae

XML Treatment for Pseudoentrophospora

XML Treatment for Pseudoentrophospora kesseensis

XML Treatment for Endogonomycetes

XML Treatment for Hoforsales

XML Treatment for Hoforsaceae

XML Treatment for Hoforsa

XML Treatment for Hoforsa rebekkae

XML Treatment for Kahvenales

XML Treatment for Kahvenaceae

XML Treatment for Kahvena

XML Treatment for Kahvena rebeccae

XML Treatment for Kelottijaerviales

XML Treatment for Kelottijaerviaceae

XML Treatment for Kelottijaervia

XML Treatment for Kelottijaervia shannonae

XML Treatment for Kungsaengenales

XML Treatment for Kungsaengenaceae

XML Treatment for Kungsaengena

XML Treatment for Kungsaengena shadiae

XML Treatment for Langduoales

XML Treatment for Langduoaceae

XML Treatment for Langduoa

XML Treatment for Langduoa dianae

XML Treatment for Lehetuales

XML Treatment for Lehetuaceae

XML Treatment for Lehetua

XML Treatment for Lehetua indrekii

XML Treatment for Lokrumales

XML Treatment for Lokrumaceae

XML Treatment for Lokruma

XML Treatment for Lokruma stenii

XML Treatment for Moosteales

XML Treatment for Moosteaceae

XML Treatment for Moostea

XML Treatment for Moostea stephanieae

XML Treatment for Nikkaluoktales

XML Treatment for Nikkaluoktaceae

XML Treatment for Nikkaluokta

XML Treatment for Nikkaluokta mahdiehiae

XML Treatment for Parniguales

XML Treatment for Parniguaceae

XML Treatment for Parnigua

XML Treatment for Parnigua craigii

XML Treatment for Riederbergales

XML Treatment for Riederbergaceae

XML Treatment for Riederberga

XML Treatment for Riederberga sylviae

XML Treatment for Ruuales

XML Treatment for Ruuaceae

XML Treatment for Ruua

XML Treatment for Ruua coralieae

XML Treatment for Tammsaareales

XML Treatment for Tammsaareaceae

XML Treatment for Tammsaarea

XML Treatment for Tammsaarea vivikae

XML Treatment for Unemaeeales

XML Treatment for Unemaeeaceae

XML Treatment for Unemaeea

XML Treatment for Unemaeea nathalieae

XML Treatment for Bifiguratales

XML Treatment for Bifigurataceae

XML Treatment for Densosporales

XML Treatment for Densosporaceae

XML Treatment for Planticonsortiaceae

XML Treatment for Endogonales

XML Treatment for Endogonaceae

XML Treatment for Jimgerdemanniaceae

XML Treatment for Vinositunicaceae

## ﻿Additional information

### Author ORCIDs

Franco Magurno https://orcid.org/0000-0002-3117-8149

Saad Alkahtani https://orcid.org/0000-0001-7381-5110

Vladimir Mikryukov https://orcid.org/0000-0003-2786-2690

### Data availability

All of the data that support the findings of this study are available in the main text or Supplementary Information.

## Supplementary materials

Supplementary material 1 Maximum Likelihood phylogram indicating phylogenetic relationships amongst *Glomeromycota* based on SSU-ITS-LSU sequences Data type pdf https://binary.pensoft.net/file/1111405This dataset is made available under the Open Database License (http://opendatacommons.org/licenses/odbl/1.0/). The Open Database License (ODbL) is a license agreement intended to allow users to freely share, modify, and use this Dataset while maintaining this same freedom for others, provided that the original source and author(s) are credited.Leho Tedersoo, Franco Magurno, Saad Alkahtani, Vladimir Mikryukov

Supplementary material 2 Maximum Likelihood phylogram indicating phylogenetic relationships amongst Gigasporales based on LSU sequences Data type pdf https://binary.pensoft.net/file/1111406This dataset is made available under the Open Database License (http://opendatacommons.org/licenses/odbl/1.0/). The Open Database License (ODbL) is a license agreement intended to allow users to freely share, modify, and use this Dataset while maintaining this same freedom for others, provided that the original source and author(s) are credited.Leho Tedersoo, Franco Magurno, Saad Alkahtani, Vladimir Mikryukov

Supplementary material 3 Maximum Likelihood phylogram indicating phylogenetic relationships amongst *Endogonomycetes* based on SSU-5.8S-LSU sequences Data type pdf https://binary.pensoft.net/file/1111407This dataset is made available under the Open Database License (http://opendatacommons.org/licenses/odbl/1.0/). The Open Database License (ODbL) is a license agreement intended to allow users to freely share, modify, and use this Dataset while maintaining this same freedom for others, provided that the original source and author(s) are credited.Leho Tedersoo, Franco Magurno, Saad Alkahtani, Vladimir Mikryukov

Supplementary material 4 Maximum Likelihood phylogram indicating phylogenetic relationships amongst *Endogonomycetes* based on SSU sequences Data type pdf https://binary.pensoft.net/file/1111408This dataset is made available under the Open Database License (http://opendatacommons.org/licenses/odbl/1.0/). The Open Database License (ODbL) is a license agreement intended to allow users to freely share, modify, and use this Dataset while maintaining this same freedom for others, provided that the original source and author(s) are credited.Leho Tedersoo, Franco Magurno, Saad Alkahtani, Vladimir Mikryukov

Supplementary material 5 Currently recognised orders, families and genera and proposed taxonomic groups in *Glomeromycota* Data type pdf https://binary.pensoft.net/file/1111409This dataset is made available under the Open Database License (http://opendatacommons.org/licenses/odbl/1.0/). The Open Database License (ODbL) is a license agreement intended to allow users to freely share, modify, and use this Dataset while maintaining this same freedom for others, provided that the original source and author(s) are credited.Leho Tedersoo, Franco Magurno, Saad Alkahtani, Vladimir Mikryukov

Supplementary material 6 Currently recognised orders, families and genera and proposed taxonomic groups in *Endogonomycetes* Data type pdf https://binary.pensoft.net/file/1111410This dataset is made available under the Open Database License (http://opendatacommons.org/licenses/odbl/1.0/). The Open Database License (ODbL) is a license agreement intended to allow users to freely share, modify, and use this Dataset while maintaining this same freedom for others, provided that the original source and author(s) are credited.Leho Tedersoo, Franco Magurno, Saad Alkahtani, Vladimir Mikryukov
