# Supplementary figures and images for: ﻿Phylogenetic classification of arbuscular mycorrhizal fungi: new species and higher-ranking taxa in Glomeromycota and Mucoromycota (class Endogonomycetes)
Source: MycoKeys. 2024 Aug 9;107:273–325. doi: 10.3897/mycokeys.107.125549 (PMC11336396; doi:10.3897/mycokeys.107.125549)

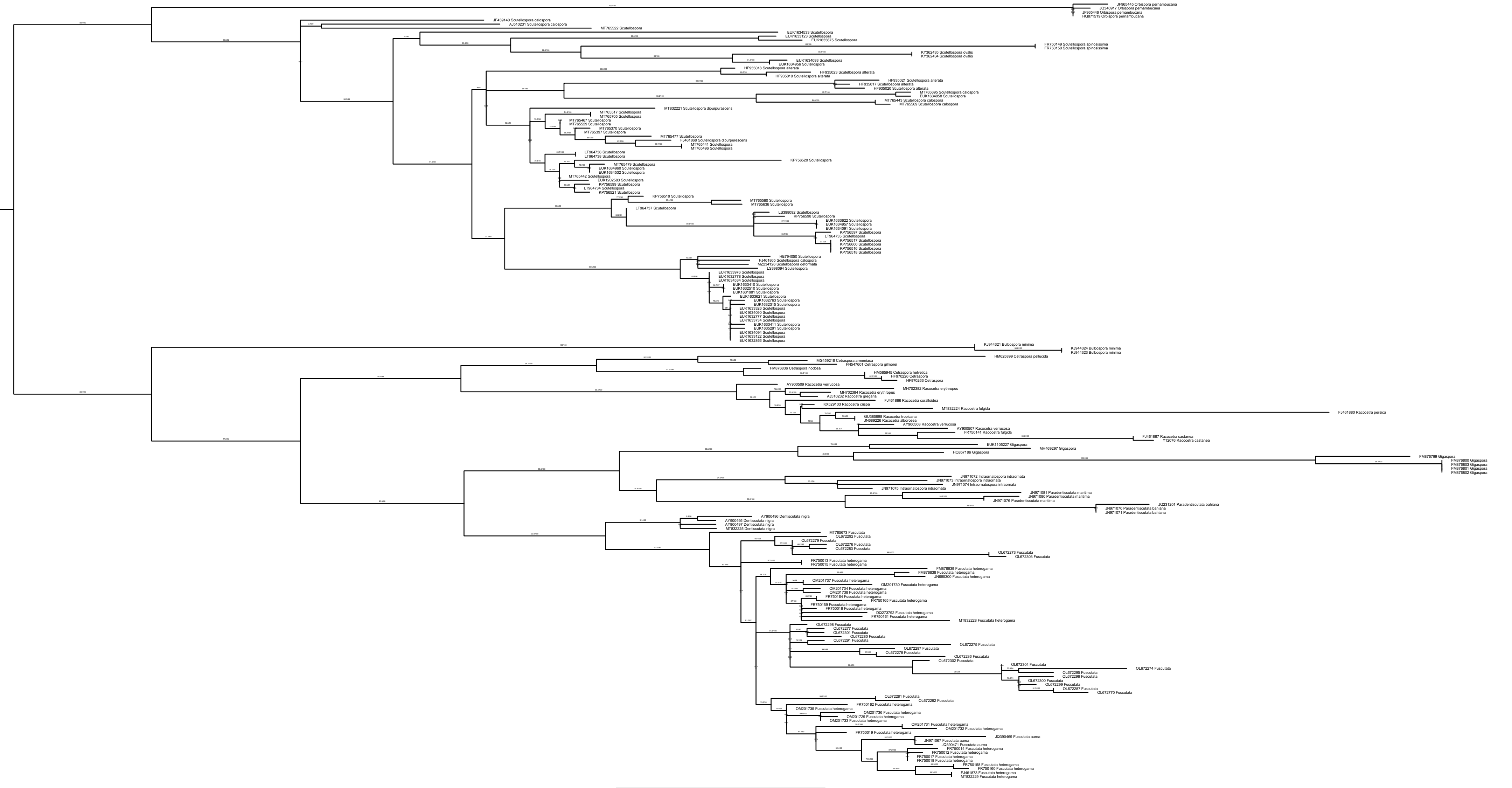

Supplement: Supplementary material 2 — Maximum Likelihood phylogram indicating phylogenetic relationships amongst Gigasporales based on LSU sequences [file mycokeys-107-273-s002.pdf]

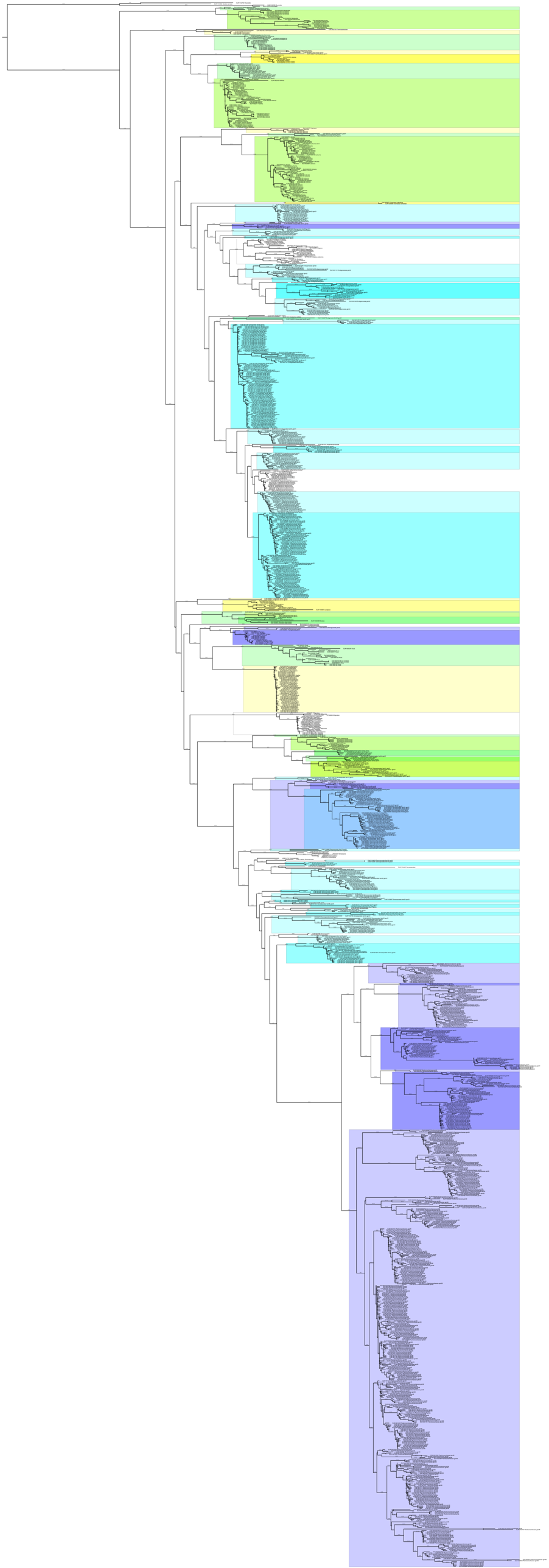

Supplement: Supplementary material 3 — Maximum Likelihood phylogram indicating phylogenetic relationships amongst Endogonomycetes based on SSU-5.8S-LSU sequences [file mycokeys-107-273-s003.pdf]

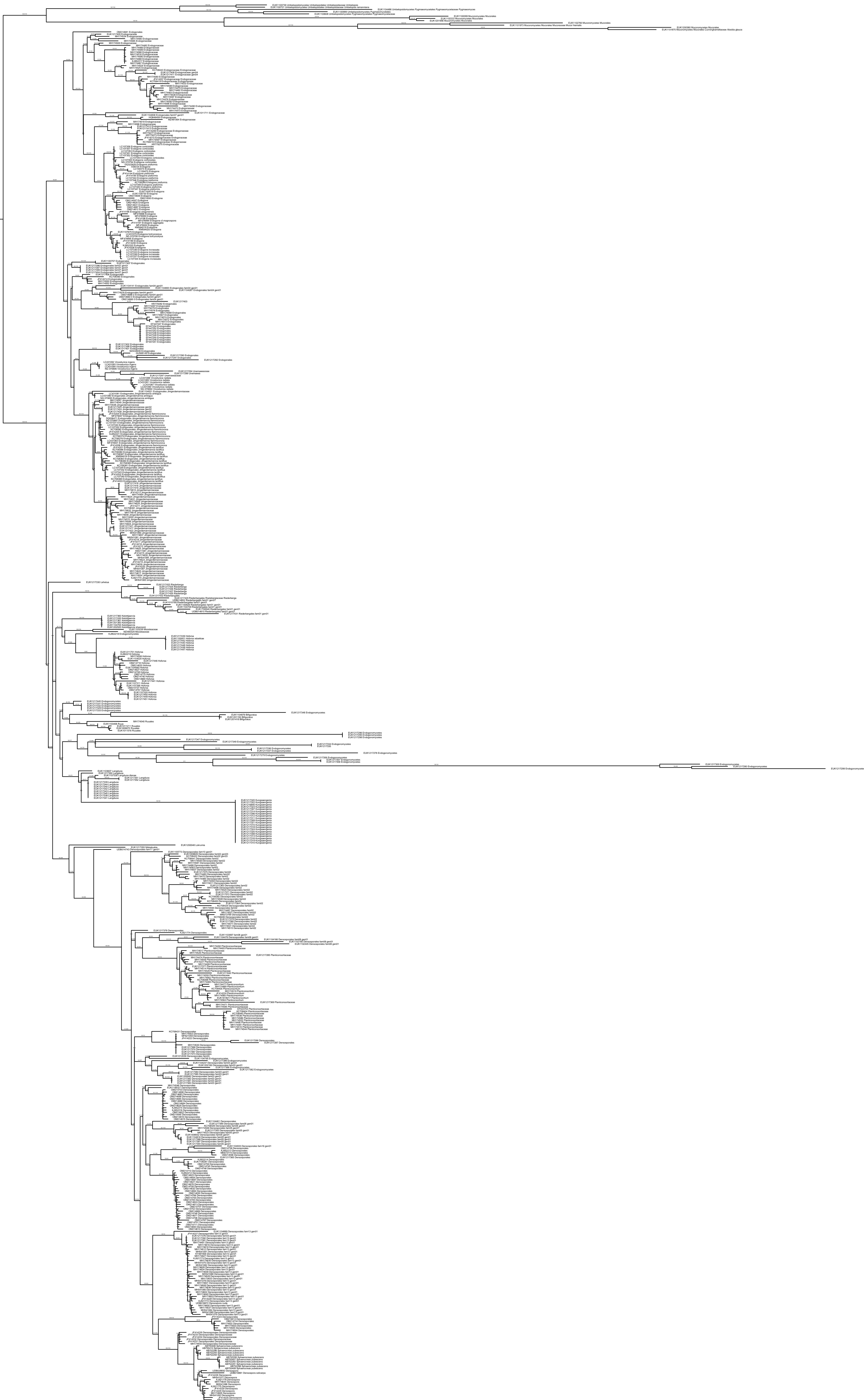

Supplement: Supplementary material 4 — Maximum Likelihood phylogram indicating phylogenetic relationships amongst Endogonomycetes based on SSU sequences [file mycokeys-107-273-s004.pdf]
