# Supplementary material for: ﻿Phylogenetic classification of arbuscular mycorrhizal fungi: new species and higher-ranking taxa in Glomeromycota and Mucoromycota (class Endogonomycetes)
Source: MycoKeys. 2024 Aug 9;107:273–325. doi: 10.3897/mycokeys.107.125549 (PMC11336396; doi:10.3897/mycokeys.107.125549)
Supplement: Supplementary material 5 — Currently recognised orders, families and genera and proposed taxonomic groups in Glomeromycota [file mycokeys-107-273-s005.pdf]

Table S1. Currently recognised orders, families and genera and proposed taxonomic groups in *Glomeromycota*.

Order	Family	Genus
Archaeosporales	*Ambisporaceae*	*Ambispora* C.Walker, Vestberg & A.Schüssler
Archaeosporales	*Archaeosporaceae*	*Archaeospora* J.B.Morton & D.Redecker
Archaeosporales	*Geosiphonaceae*	*Geosiphon* F.Wettst.
Archaeosporales	*Polonosporaceae*	*Polonospora* Błaszk., Niezgoda, B.T.Goto & Magurno
Archaeosporales	*Archaeosporaceae*	*Archaeosporaceae*_gen01
Archaeosporales	*Archaeosporaceae*	*Archaeosporaceae*_gen02
Diversisporales	*Acaulosporaceae*	*Acaulospora* Gerd. & Trappe
Diversisporales	*Diversisporaceae*	*Desertispora* Błaszk., Kozłowska, Ryszka, Al-Yahya'ei & Symanczik
Diversisporales	*Diversisporaceae*	*Diversispora* C.Walker & A.Schüßler
Diversisporales	*Diversisporaceae*	*Diversisporaceae*_gen01
Diversisporales	*Diversisporaceae*	*Diversisporaceae*_gen02
Diversisporales	*Diversisporaceae*	*Redeckera* C.Walker & A.Schüßler
Diversisporales	*Diversisporaceae*	*Sieverdingia* Błaszk., Niezgoda & B.T.Goto
Diversisporales	*Pacisporaceae*	*Pacispora* Sieverd. & Oehl
Diversisporales	*Sacculosporaceae*	*Sacculospora* Oehl, Sieverd., G.A.Silva, B.T.Goto, I.C.Sánchez & Palenzuela
Entrophosporales	*Entrophosporaceae*	*Entrophospora* Oehl, Sieverd., G.A.Silva, B.T.Goto, I.C.Sánchez & Palenzuela
Entrophosporales	*Pseudoentrophosporaceae*	*Pseudoentrophospora* Tedersoo & Magurno

Gigasporales	*Gigasporaceae*	*Bulbospora* Oehl & G.A.Silva
Gigasporales	*Dentiscutataceae*	*Dentiscutata* Sieverd., F.A.Souza & Oehl
Gigasporales	*Dentiscutataceae*	*Fuscutata* Oehl, F.A.Souza & Sieverd.
Gigasporales	*Gigasporaceae*	*Gigaspora* Gerd. & Trappe
Gigasporales	*Intraornatosporaceae*	*Intraornatospora* B.T.Goto, Oehl & G.A.Silva
Gigasporales	*Intraornatosporaceae*	*Paradentiscutata* B.T.Goto, Oehl & G.A.Silva
Gigasporales	*Racocetraceae*	*Cetraspora* Oehl, F.A.Souza & Sieverd.
Gigasporales	*Racocetraceae*	*Racocetra* Oehl, F.A.Souza & Sieverd.
Gigasporales	*Scutellosporaceae*	*Scutellospora* C.Walker & F.E.Sanders
Gigasporales	*Scutellosporaceae*	*Orbispora* Oehl, G.A.Silva & D.K.Silva
Glomerales	*Glomeraceae*	*Complexispora* Błaszk., B.T.Goto, Niezgoda & Magurno
Glomerales	*Glomeraceae*	*Dominikia* Błaszk., Chwat & Kovács
Glomerales	*Glomeraceae*	*Epigeocarpum* Błaszk., B.T.Goto, Jobim, Niezgoda & Marguno
Glomerales	*Glomeraceae*	*Funneliformis* C.Walker & A.Schüßler
Glomerales	*Glomeraceae*	*Funneliglomus* Corazon-Guivin, G.A.Silva & Oehl
Glomerales	*Glomeraceae*	*Glomeraceae*_gen01
Glomerales	*Glomeraceae*	*Glomeraceae*_gen02
Glomerales	*Glomeraceae*	*Glomeraceae*_gen03
Glomerales	*Glomeraceae*	*Glomeraceae*_gen04

Glomerales	*Glomeraceae*	*Glomeraceae*_gen05
Glomerales	*Glomeraceae*	*Glomeraceae*_gen06
Glomerales	*Glomeraceae*	*Glomeraceae*_gen07
Glomerales	*Glomeraceae*	*Glomeraceae*_gen08
Glomerales	*Glomeraceae*	*Glomeraceae*_gen09
Glomerales	*Glomeraceae*	*Glomeraceae*_gen10
Glomerales	*Glomeraceae*	*Glomeraceae*_gen11
Glomerales	*Glomeraceae*	*Glomeraceae*_gen12
Glomerales	*Glomeraceae*	*Glomeraceae*_gen13
Glomerales	*Glomeraceae*	*Glomeraceae*_gen14
Glomerales	*Glomeraceae*	*Glomeraceae*_gen15
Glomerales	*Glomeraceae*	*Glomeraceae*_gen16
Glomerales	*Glomeraceae*	*Glomeraceae*_gen17
Glomerales	*Glomeraceae*	*Glomeraceae*_gen18
Glomerales	*Glomeraceae*	*Glomeraceae*_gen19
Glomerales	*Glomeraceae*	*Glomeraceae*_gen20
Glomerales	*Glomeraceae*	*Glomeraceae*_gen21
Glomerales	*Glomeraceae*	*Glomeraceae*_gen22
Glomerales	*Glomeraceae*	*Glomeraceae*_gen23

Glomerales	*Glomeraceae*	*Glomeraceae*_gen24
Glomerales	*Glomeraceae*	*Glomeraceae*_gen25
Glomerales	*Glomeraceae*	*Glomeraceae*_gen26
Glomerales	*Glomeraceae*	*Glomeraceae*_gen27
Glomerales	*Glomeraceae*	*Glomeraceae*_gen28
Glomerales	*Glomeraceae*	*Glomeraceae*_gen29
Glomerales	*Glomeraceae*	*Glomeraceae*_gen30
Glomerales	*Glomeraceae*	*Glomus* Tul. & C.Tul.
Glomerales	*Glomeraceae*	*Halonatospora* Błaszk., Niezgoda, B.T.Goto & Kozłowska
Glomerales	*Glomeraceae*	*Kamienskia* Błaszk., Chwat & Kovács
Glomerales	*Glomeraceae*	*Microdominikia* Oehl, Corazon-Guivin & G.A.Silva
Glomerales	*Glomeraceae*	*Microkamienskia* Corazon-Guivin, G.A.Silva & Oehl
Glomerales	*Glomeraceae*	*Nanoglomus* Corazon-Guivin, G.A.Silva & Oehl
Glomerales	*Glomeraceae*	*Oehlia* Błaszk., Kozłowska, Niezgoda, B.T.Goto & Dalpé
Glomerales	*Glomeraceae*	*Orientoglomus* G.A.Silva, Oehl & Corazon-Guivin
Glomerales	*Glomeraceae*	*Parvocarpum* Magurno
Glomerales	*Glomeraceae*	*Rhizoglomus* Sieverd., G.A.Silva & Oehl
Glomerales	*Glomeraceae*	*Sclerocarpum* B.T.Goto, Błaszk., Niezgoda, A.Kozłowska & Jobim
Glomerales	*Glomeraceae*	*Sclerocystis* Berk. & Broome

Glomerales	*Glomeraceae*	*Septoglomus* Sieverd., G.A.Silva & Oehl
Glomerales	*Glomeraceae*	*Silvaspora* Błaszk., Niezgoda, B.T.Goto, Crossay & Magurno
Glomerales	*Glomeraceae*	*Viscospora* Sieverd., Oehl & G.A.Silva
Paraglomerales	*Paraglomeraceae*	*Paraglomeraceae*_gen01
Paraglomerales	*Paraglomeraceae*	*Innospora* Błaszk., Kovács, Chwat & Kozłowska
Paraglomerales	*Paraglomeraceae*	*Paraglomus* J.B.Morton & D.Redecker
Paraglomerales	*Pervetustaceae*	*Pervetustus* Błaszk., Chwat, Kozłowska, Symanczik & Al-Yahya'ei
