# Supplementary material for: ﻿Phylogenetic classification of arbuscular mycorrhizal fungi: new species and higher-ranking taxa in Glomeromycota and Mucoromycota (class Endogonomycetes)
Source: MycoKeys. 2024 Aug 9;107:273–325. doi: 10.3897/mycokeys.107.125549 (PMC11336396; doi:10.3897/mycokeys.107.125549)
Supplement: Supplementary material 6 — Currently recognised orders, families and genera and proposed taxonomic groups in Endogonomycetes [file mycokeys-107-273-s006.pdf]

Table S2. Currently recognised orders, families and genera and proposed taxonomic groups in *Endogonomycetes*.

Order	Family	Genus
Densosporales	*Densosporaceae*	*Densospora* McGee
Densosporales	*Densosporales* fam01	*Densosporales* fam01 gen01
Densosporales	*Densosporales* fam02	*Densosporales* fam02 gen01
Densosporales	*Densosporales* fam02	*Densosporales* fam02 gen02
Densosporales	*Densosporales* fam02	*Densosporales* fam02 gen03
Densosporales	*Densosporales* fam03	*Densosporales* fam03 gen01
Densosporales	*Densosporales* fam04	*Densosporales* fam04 gen01
Densosporales	*Densosporales* fam05	*Densosporales* fam05 gen01
Densosporales	*Densosporales* fam06	*Densosporales* fam06 gen01
Densosporales	*Densosporales* fam07	*Densosporales* fam07 gen01
Densosporales	*Densosporales* fam08	*Densosporales* fam08 gen01
Densosporales	*Densosporales* fam09	*Densosporales* fam09 gen01
Densosporales	*Densosporales* fam10	*Densosporales* fam10 gen01

Densosporales	*Densosporales* fam11	*Densosporales* fam11 gen01
Densosporales	*Densosporales* fam12	*Densosporales* fam12 gen01
Densosporales	*Densosporales* fam13	*Densosporales* fam13 gen01
Densosporales	*Densosporales* fam14	*Densosporales* fam14 gen01
Densosporales	*Densosporales* fam15	*Densosporales* fam15 gen01
Densosporales	*Densosporales* fam16	*Densosporales* fam16 gen01
Densosporales	*Planticonsortiaceae*	*Planticonsortiaceae* gen01
Densosporales	*Planticonsortiaceae*	*Planticonsortiaceae* gen02
Densosporales	*Planticonsortiaceae*	*Planticonsortiaceae* gen03
Densosporales	*Planticonsortiaceae*	*Planticonsortiaceae* gen04
Densosporales	*Planticonsortiaceae*	*Planticonsortiaceae* gen05
Densosporales	*Planticonsortiaceae*	*Planticonsortiaceae* gen06
Densosporales	*Planticonsortiaceae*	*Planticonsortiaceae* gen07
Densosporales	*Planticonsortiaceae*	*Planticonsortium* C.Walker & D.Redecker

Endogonales	*Endogonaceae*	*Endogonaceae* gen01
Endogonales	*Endogonaceae*	*Endogonaceae* gen02
Endogonales	*Endogonaceae*	*Endogonaceae* gen03
Endogonales	*Endogonaceae*	*Endogonaceae* gen04
Endogonales	*Endogonaceae*	*Endogone* Link
Endogonales	*Endogonales* fam i.sedis	*Peridiospora* C.G.Wu & Suh J.Lin
Endogonales	*Endogonales* fam i.sedis	*Sclerogone* Warcup
Endogonales	*Endogonales* fam01	*Endogonales* fam01 gen01
Endogonales	*Endogonales* fam01	*Endogonales* fam01 gen02
Endogonales	*Endogonales* fam02	*Endogonales* fam02 gen01
Endogonales	*Endogonales* fam03	*Endogonales* fam03 gen01
Endogonales	*Endogonales* fam04	*Endogonales* fam04 gen01
Endogonales	*Endogonales* fam05	*Endogonales* fam05 gen01
Endogonales	*Endogonales* fam06	*Endogonales* fam06 gen01

Endogonales	*Endogonales* fam06	*Endogonales* fam07 gen01
Endogonales	*Jimgerdemanniaceae*	*Jimgerdemannia* Trappe, Desirò, M.E.Sm., Bonito & Bidartondo
Endogonales	*Jimgerdemanniaceae*	*Jimgerdemanniaceae* gen01
Endogonales	*Jimgerdemanniaceae*	*Jimgerdemanniaceae* gen02
Endogonales	*Jimgerdemanniaceae*	*Jimgerdemanniaceae* gen03
Endogonales	*Jimgerdemanniaceae*	*Jimgerdemanniaceae* gen04
Endogonales	*Jimgerdemanniaceae*	*Jimgerdemanniaceae* gen05
Endogonales	*Jimgerdemanniaceae*	*Sphaerocreas* Sacc. & Ellis
Endogonales	*Vinositunicaceae*	*Vinositunica* Koh.Yamam., Degawa & A.Yamada
Hoforsales	*Hoforsaceae*	*Hoforsa* Tedersoo
Hoforsales	*Hoforsales* fam01	*Hoforsales* fam01 gen01
Kahvenales	*Kahvenaceae*	*Kahvena* Tedersoo
Kelottijaerviales	*Kelottijaerviaceae*	*Kelottijaervia* Tedersoo
Kungsaengenales	*Kungsaengenaceae*	*Kungsaengena* Tedersoo
Langduoales	*Langduoaceae*	*Langduoa* Tedersoo
Langduoales	*Langduoles* fam01	*Langduoaceae* fam01 gen01

Lehetuales	*Lehetuaceae*	*Lehetua* Tedersoo
Lehetuales	*Lehetuaceae*	*Lehetuaceae* gen01
Lokrumales	*Lokrumaceae*	*Lokruma* Tedersoo
Lokrumales	*Lokrumales* fam01	*Lokrumales* fam01 gen01
Moosteales	*Moosteaceae*	*Moostea* Tedersoo
Moosteales	*Moosteaceae*	*Moosteaceae* gen01
Moosteales	*Moosteaceae*	*Moosteaceae* gen02
Nikkaluoktales	*Nikkaluoktaceae*	*Nikkaluokta* Tedersoo
Nikkaluoktales	*Nikkaluoktaceae*	*Nikkaluoktaceae* gen01
Parniguales	*Parniguaceae*	*Parnigua* Tedersoo
Parniguales	*Parniguaceae*	*Parniguaceae* gen01
Riederbergales	*Riederbergaceae*	*Riederberga* Tedersoo
Riederbergales	*Riederbergales* fam01	*Riederbergales* fam01 gen01
Riederbergales	*Riederbergales* fam02	*Riederbergales* fam02 gen01
Riederbergales	*Riederbergales* fam03	*Riederbergales* fam03 gen01
Riederbergales	*Riederbergales* fam04	*Riederbergales* fam04 gen01
Riederbergales	*Riederbergales* fam05	*Riederbergales* fam05 gen01

Ruuales	*Ruuaceae*	*Ruua* Tedersoo
Ruuales	*Ruuaceae*	*Ruuaceae* gen01
Tammsaareales	*Tammsaareaceae*	*Tammsaarea* Tedersoo
Unemaeeales	*Unemaeeaceae*	*Unemaeea* Tedersoo
