# Supplementary material for: ﻿Phylogenetic classification of arbuscular mycorrhizal fungi: new species and higher-ranking taxa in Glomeromycota and Mucoromycota (class Endogonomycetes)
Source: MycoKeys. 2024 Aug 9;107:273–325. doi: 10.3897/mycokeys.107.125549 (PMC11336396; doi:10.3897/mycokeys.107.125549)

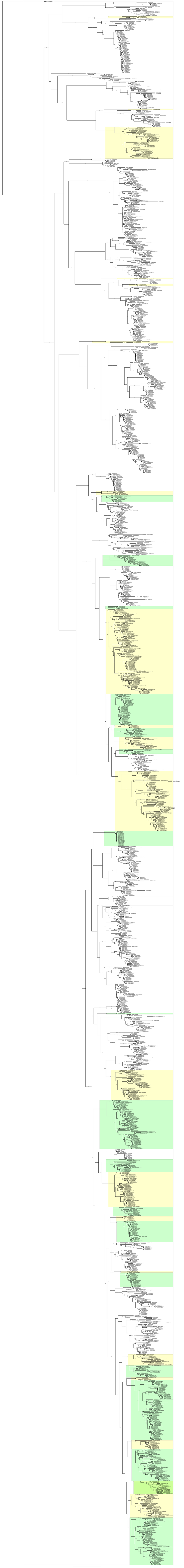

Supplement: Supplementary material 1 — Maximum Likelihood phylogram indicating phylogenetic relationships amongst Glomeromycota based on SSU-ITS-LSU sequences [file mycokeys-107-273-s001.pdf]
